# Nuclear Localised MORE SULPHUR ACCUMULATION1 Epigenetically Regulates Sulphur Homeostasis in *Arabidopsis thaliana*

**DOI:** 10.1371/journal.pgen.1006298

**Published:** 2016-09-13

**Authors:** Xin-Yuan Huang, Dai-Yin Chao, Anna Koprivova, John Danku, Markus Wirtz, Steffen Müller, Francisco J. Sandoval, Hermann Bauwe, Sanja Roje, Brian Dilkes, Rüdiger Hell, Stanislav Kopriva, David E Salt

**Affiliations:** 1 Institute of Biological and Environmental Sciences, University of Aberdeen, Aberdeen, United Kingdom; 2 Botanical Institute, Cluster of Excellence on Plant Sciences (CEPLAS), University of Cologne, Cologne, Germany; 3 Centre for Organismal Studies Heidelberg, Heidelberg University, Heidelberg, Germany; 4 Department of Plant Physiology, University of Rostock, Rostock, Germany; 5 Institute of Biological Chemistry, Washington State University, Pullman, Washington, United States of America; 6 Department of Biochemistry, Purdue University, West Lafayette, Indiana, United States of America; Rutgers University, UNITED STATES

## Abstract

Sulphur (S) is an essential element for all living organisms. The uptake, assimilation and metabolism of S in plants are well studied. However, the regulation of S homeostasis remains largely unknown. Here, we report on the identification and characterisation of the *more sulphur accumulation1* (*msa1-1*) mutant. The MSA1 protein is localized to the nucleus and is required for both S-adenosylmethionine (SAM) production and DNA methylation. Loss of function of the nuclear localised MSA1 leads to a reduction in SAM in roots and a strong S-deficiency response even at ample S supply, causing an over-accumulation of sulphate, sulphite, cysteine and glutathione. Supplementation with SAM suppresses this high S phenotype. Furthermore, mutation of *MSA1* affects genome-wide DNA methylation, including the methylation of S-deficiency responsive genes. Elevated S accumulation in *msa1-1* requires the increased expression of the sulphate transporter genes *SULTR1;1* and *SULTR1;2* which are also differentially methylated in *msa1-1*. Our results suggest a novel function for MSA1 in the nucleus in regulating SAM biosynthesis and maintaining S homeostasis epigenetically via DNA methylation.

## Author Summary

Sulphur is an essential element for all living organisms including plants. Plants take up sulphur from the soil mainly in the form of inorganic sulphate. The uptake of sulphate and assimilation of sulphur have been well studied. However, the regulation of sulphur accumulation in plants remains largely unknown. In this study, we characterize the high leaf sulphur mutant *more sulphur accumulation1* (*msa1-1*) and demonstrate the function of *MSA1* in controlling sulphur accumulation in *Arabidopsis thaliana*. The MSA1 protein is localized to the nucleus and is required for the biosynthesis of S-adenosylmethionine (SAM) which is a universal methyl donor for many methylation reactions, including DNA methylation. Loss of function of *MSA1* reduces the SAM level in roots and affects genome-wide DNA methylation, including the methylation of sulphate transporter genes. We show that the high sulphur phenotype of *msa1-1* requires elevated expression of the sulphate transporter genes which are differentially methylated in *msa1-1*. Our results suggest a connection between sulphur homeostasis and DNA methylation that is mediated by MSA1.

## Introduction

Sulphur (S) is one of the essential macronutrients required for plant growth and plays a pivotal role in plant development and metabolism. Plants take up S in the form of inorganic sulphate from the rhizosphere mainly by two high-affinity sulphate transporters, SULTR1;1 and SULTR1;2 [1–3]. Before reduction, sulphate is first activated by ATP sulfurylase (ATPS) to adenosine 5′-phosphosulfate (APS) [4, 5]. APS is either reduced by APS reductase (APR) to sulphite, or phosphorylated by APS kinase (APK) to form 3′-phosphoadenosine 5′-phosphosulfate (PAPS) which provides activated sulphate for many sulphation reactions. In the primary sulphate assimilation branch, sulphite is further reduced to sulphide by sulphite reductase (SiR). Subsequently, *O*-acetylserine (thiol) lyase (OAS-TL) catalyzes the condensation of sulphide and *O*-acetylserine (OAS) to form cysteine (Cys), the first organic-reduced sulphur compound. Cys then serves as a precursor for the biosynthesis of methionine (Met), glutathione (GSH), vitamins and other sulphur derivatives. Met can be further used for the biosynthesis of S-adenosylmethionine (SAM) which is a universal methyl group donor for many methylation reactions [6], suggesting a potential yet unexplored connection between S metabolism and methylation reactions, including DNA methylation.

Compared to the well-characterized sulphate uptake and S assimilation pathway [4, 5], our knowledge of the regulation of S homeostasis in plants is limited. The transcription factor SLIM1 (SULFUR LIMITATION 1) acts as a central transcriptional regulator which controls sulphate uptake and the balance of global sulphur utilization under S deficiency by regulating the expression of *SULTR1;1* and *SULTR1;2* and genes involved in the degradation of glucosinolates [7]. Another regulator involved in S starvation response is miR395. miR395 targets to *ATPS1*, *ATPS4* and the low-affinity sulphate transporter gene *SULTR2;1* and regulates their expression [8, 9]. miR395 is strongly induced by S deficiency and regulates the translocation of sulphate from old to young leaves as well as from roots to shoots under sulphate limited conditions [10, 11]. The induction of miR395 by S deficiency is controlled by SLIM1 and thus SLIM1 and miR395 are two important components of the regulatory circuit controlling plant sulphate assimilation in S deficient conditions [8, 11]. The expression of *SULTR1;1* and *SULTR1;2* is controlled by SLIM1 [7], while *APR1* and *APR2* are controlled by the transcriptional factor LONG HYPOCOTYL5 (HY5) [12]. Unlike *SULTR1;1* and *SULTR1;2*, which are induced by S deficiency in both shoots and roots [2], *SULTR2;1* shows the opposite response to S deficiency in shoots and roots, with decreased expression in shoots but strong induction in roots [1]. The repression of *SULTR2;1* in shoots is consistent with the upregulation of miR395 under S deficiency, which targets to *SULTR2;1* mRNA and suppresses its expression [8]. However, both *SULTR2;1* and miR395 are upregulated in roots under S deficiency. This is due to their cell-type-specific expressions in roots, in which miR395 only expresses in the phloem companion cells and is unable to target the *SULTR2;1* mRNA in xylem parenchyma and pericycle cells [8]. Several *cis*-acting elements responsive to S deficiency have been identified, such as sulphur-responsive element (SURE) in the promoter of *SULTR1;1* [13], and SURE21A and SURE21B in the 3’-untranslated region of *SULTR2;1* [14]. However, the transcription factors targeting these *cis*-acting elements have not been identified.

Regulation of gene expression at the transcriptional and posttranscriptional level is known to play critical roles in the way plants respond to environmental stresses. Recent studies have suggested that epigenetic regulation of gene expression also plays an important role in these responses [15, 16]. DNA methylation is one of the most studied epigenetic modifications, in which the methyl group from SAM is transferred to the 5’ position of a cytosine to form 5-methylcytosine. In plants, DNA methylation occurs in the three different sequence contexts CG, CHG and CHH (where H is A, C or T), through different pathways [17]. DNA methylation is involved in genomic imprinting, silencing of the expression of genes and transposons, and regulating gene expression under environmental stresses, including in response to nutrient status [15, 16]. Dynamic DNA methylation changes have been recently shown to modulate the expression of genes in response to phosphorus starvation in *A*. *thaliana* [18] and in rice [19]. However, the involvement of altered DNA methylation in response to other nutrient deficiencies is not clear.

In this study, we describe the identification and characterization of the *more sulphur accumulation1-1* (*msa1-1*) mutant in *A*. *thaliana* which has high leaf S. We propose that MSA1 functions in the nucleus to maintain DNA methylation including that required for epigenetic regulation of sulphur-homeostasis through an involvement in the maintenance of SAM levels.

## Results

### Identification of *A*. *thaliana msa1-1* mutant

**Fig 1:**
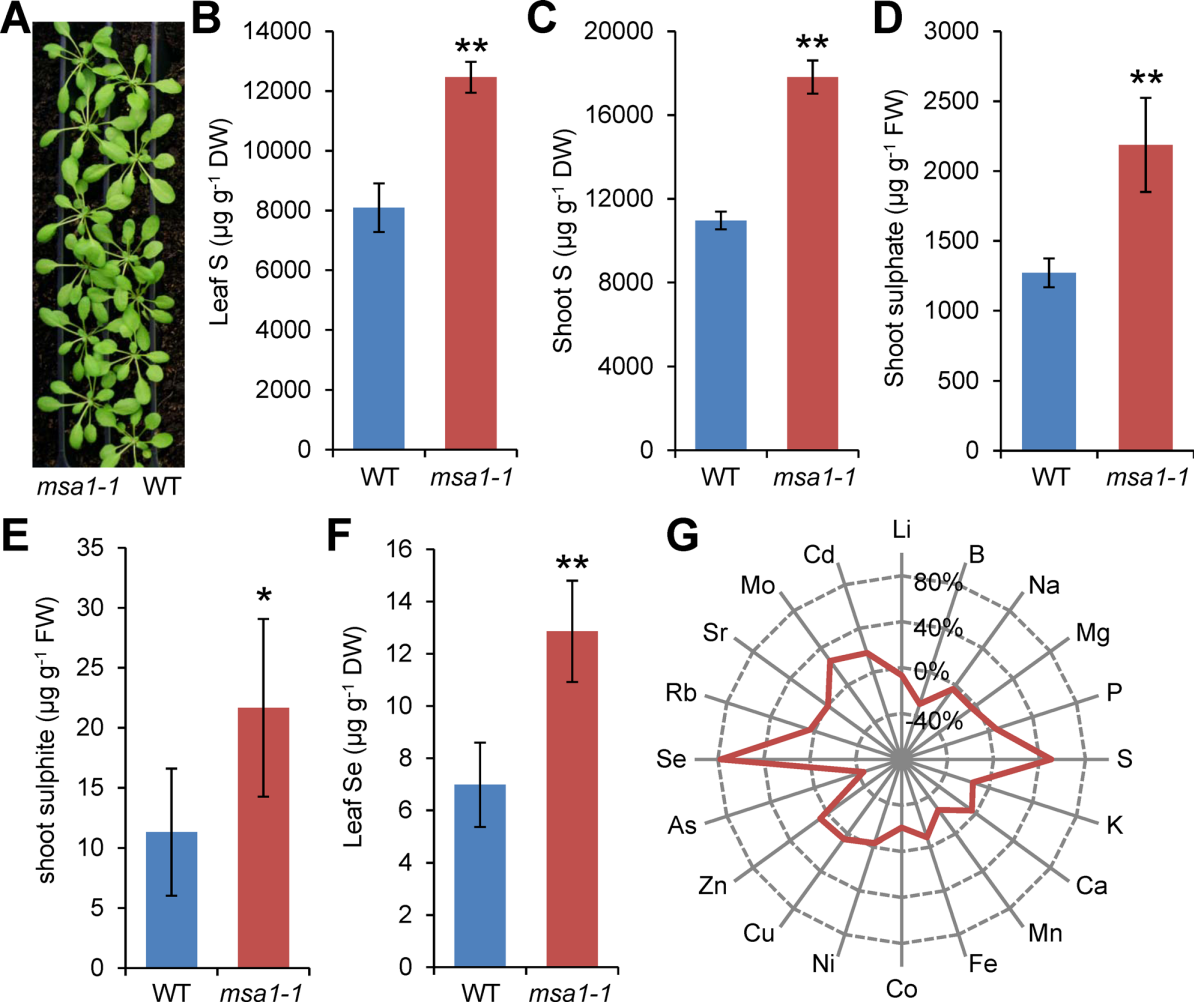
High sulphur phenotype of *msa1-1*. (A) Five-week-old WT (right row) and *msa1-1* mutant (left row) plants grown in soil. The picture was taken before harvesting samples for ICP-MS. (B) Total sulphur in the leaf of five-week-old WT and *msa1-1* mutant plants grown in soil. (C) Total sulphur in the shoot of WT and *msa1-1* mutant plants grown on agar solidified MGRL media. (D) Sulphate content in the shoot of WT and *msa1-1* mutant plants grown on agar solidified MGRL media. (E) Sulphite content in the shoot of WT and *msa1-1* mutant plants grown on agar solidified MGRL media. (F) Total Se content in the leaf of five-week old plants grown in soil. (G) Percentage difference of 20 elements of *msa1-1* mutant compared with the WT, visualized as the radar chart. Data in (B) to (F) are presented as means ± SD with *n* = 12 in (B) and (F), *n* = 6 in (C), *n* = 3 in (D) and (E). *, *P* ≤ 0.05; **, *P* ≤ 0.01, Student’s *t* test. DW, dry weight; FW, fresh weight.

In our previous search for *A*. *thaliana* mutants with altered leaf elemental composition (ionome), we identified 51 fast neutron–mutagenized mutants, and several of them have now been well characterized [20–25]. To further identify mutants with an altered leaf ionome, we conducted a screen of ethyl methanesulfonate (EMS)–mutagenized plants. Here, we describe the *msa1-1* mutant identified as containing elevated leaf S. The *msa1-1* mutant accumulated 54% higher total leaf S compared to the wild type (WT) Col-0 when grown in soil, and 63% higher when grown on agar-solidified media, without obvious visible morphological changes (Fig 1A–1C). The high S phenotype was observed only in shoots and not in roots when grown on agar-solidified media with different concentrations of sulphate (S1A and S1B Fig). Further analysis showed that both sulphate and sulphite concentrations are elevated in the shoots of *msa1-1* (Fig 1D and 1E). Of the 20 elements measured, selenium (Se) was also found to be higher in the leaves of *msa1-1* compared to WT (Fig 1F and 1G), which is likely due to the uptake of selenate by sulphate transporters in plants [26].

### Mapping the causal gene in *msa1-1*

**Fig 2:**
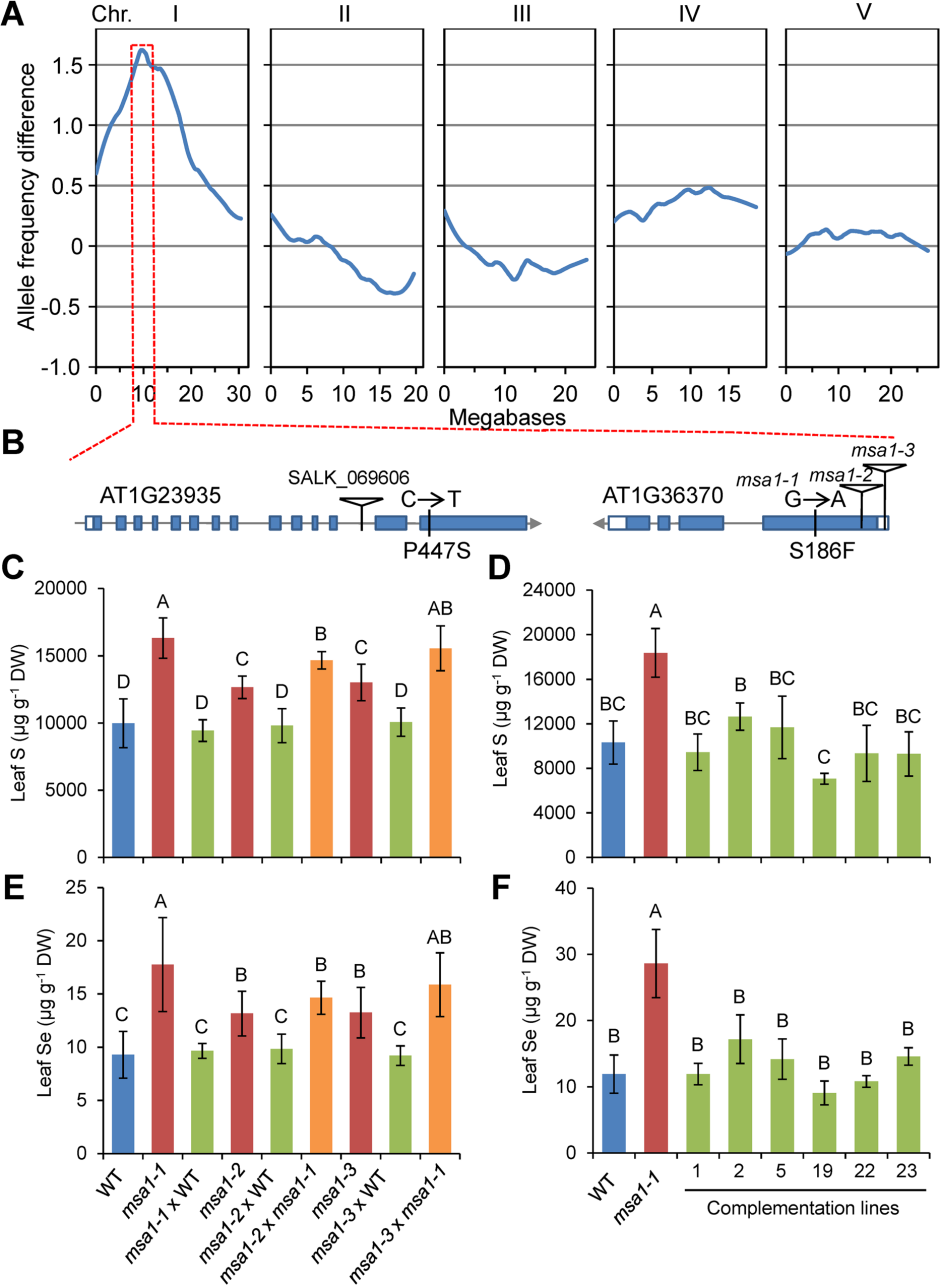
Identification the causal gene for *msa1-1*. (A) Bulk segregant analysis (BSA) of the high leaf S phenotype in an *msa1-1* × Ler-0 F2 population. Blue lines represent allele frequency differences between the pools of F2 plants with high and low leaf S (*n* = 40) at the polymorphic SNPs between Col-0 and L*er*-0. (B) Identification of genes with mutations in the BSA confidence interval identified by SOLiD sequencing. Blue bars, grey lines and white bars represent exons, introns and untranslated region, respectively. (C, E) Genetic complementation of T-DNA insertion alleles by crossing with WT Col-0 or *msa1-1*. The S (C) and Se (E) content in leaves of F1 plants were determined. Data are presented as means ± SD (*n =* 6 to 12). (D, F) Transgenic complementation of the high S phenotype of *msa1-1*. The S (D) and Se (F) content in the leaves of six independent transgenic complementation lines were determined. Data are presented as means ± SD (*n =* 3 to 12). Columns with different letters indicate significant differences (*P* ≤ 0.01, least significant difference test). DW, dry weight. ICP-MS data is accessible using the digital object identifier (DOI) 10.4231/T95Q4T1C (see http://dx.doi.org/).

Leaf S and Se accumulation in F1 plants derived from the *msa1-1* × L*er*-0 cross, as well as the segregation of the S and Se phenotype in the F2 population, revealed that *msa1-1* is a recessive mutation (S2A–S2D Fig). The causal locus was mapped to a 10 Mb interval on chromosome 1 using bulk segregant analysis (BSA; Fig 2A). Two genes with nonsynonymous mutations in the BSA mapping interval were identified by whole genome sequencing, *AT1G23935* annotated as *apoptosis inhibitory 5* and *AT1G36370* previously annotated as *serine hydroxymethyltransferase 7* (*SHM7*) based on sequence homology but without functional data [27] (Fig 2B). The C to T transition in *AT1G23935* and G to A transition in *AT1G36370* lead to P447S and S186F mutations, respectively (Fig 2B). Notably, the S^186^ amino acid residue mutated in the protein encoded by *AT1G36370* is conserved among authentic plant SHM proteins (S3A Fig). Serine hydroxymethyltransferase is a ubiquitous and conserved enzyme in living organisms from bacteria to higher plants and mammals, playing important roles in glycine-into-serine interconversion and cellular one-carbon (C_1_) folate metabolism [28–30]. As a pyridoxal-5’-phosphate (PLP) dependent enzyme, SHM catalyses the reversible conversion of serine (Ser) and tetrahydrofolate (THF) to glycine (Gly) and 5,-10-methylene-THF [31]. Homology modelling of the protein encoded by *AT1G36370* using a known SHM indicated that Y^185^ and E^187^, neighbouring amino acid residues to S^186^, form part of the binding site of SHM for the co-factor PLP and folate [32] (S3B–S3D Fig). Mutation of S^186^ to Phe is predicted to destroy the H-bonds between S^186^ and its neighbouring residues and generate steric hindrances (S3C and S3D Fig), which may affect the function of the protein encoded by *AT1G36370*.

To establish which gene is driving high S and Se in *msa1-1*, we obtained one T-DNA insertion allele for *AT1G23935* (SALK_069606) and two for *AT1G36370* (SALK_044268 and SALK_118251) (Figs 2B and S4A–S4C). The S and Se concentration in leaves of both the T-DNA alleles of *AT1G36370* were significantly higher than WT and similar to *msa1-1*, while no changes were observed for the *AT1G23935* T-DNA allele, indicating *AT1G36370* is likely the causal gene (S4D and S4E Fig). To further establish that *AT1G36370* is the causal gene, we crossed SALK_044268 (designated *msa1-2*) and SALK_118251 (designated *msa1-3*) with Col-0 WT and with *msa1-1*. All F1 plants from the *msa1-1* × Col-0 WT, *msa1-2* × Col-0 WT and *msa1-3* × Col-0 WT crosses showed similar leaf S and Se concentrations to Col-0 WT, as expected for a recessive mutation. However, the F1 progeny from the *msa1-2* × *msa1-1* and *msa1-3* × *msa1-1* crosses all contained higher leaf S and Se than Col-0 WT, indicating these mutants are allelic (Fig 2C and 2E). We further transformed the WT genomic DNA fragments of *MSA1* into *msa1-1*. Six independent T2 complementation lines all showed leaf S and Se levels similar to WT (Fig 2D and 2F). Both genetic and transgenic complementation demonstrated that *AT1G36370* is the causal gene underlying the high S and Se phenotype of *msa1-1*, and we name *AT1G36370* as *MSA1*.

### Tissue expression pattern of *MSA1* and subcellular localization of MSA1

**Fig 3:**
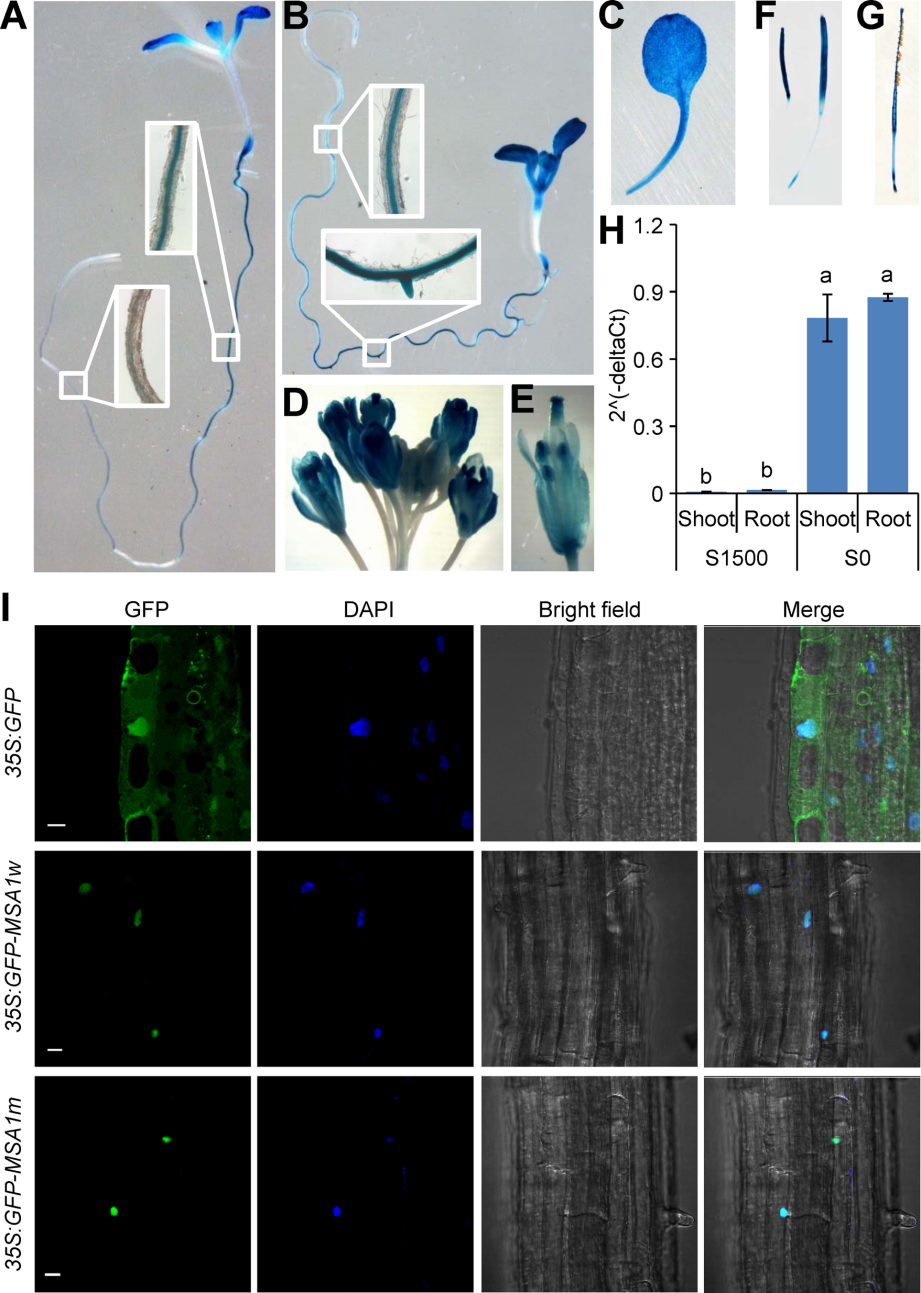
Expression pattern and subcellular localization of *MSA1*. (A-G) Histochemical GUS staining of *MSA1* promoter-GUS transgenic plants. One-week-old seedlings grown on agar solidified MGRL media with 1500 μM sulphate (A) or without sulphate (B). (C) A leaf from a two-week-old plant grown on agar solidified MGRL media with 1500μM sulphate; (D) the inflorescence of a plant grown in soil; (E) a flower; (F) developing siliques; (G) a mature silique. (H) Expression of *MSA1* was strongly induced by S-deficiency. Plants were grown in S sufficient conditions (S1500) or S deficient conditions (S0) for two weeks. Expression level of *MSA1* was normalized to the internal control gene *UBQ10*, and presented as 2^(-deltaCt) with means ± SD (*n =* 3). Columns with different letters indicate significant differences (*P* ≤ 0.01, LSD test). (I) Subcellular localization of MSA1. Constructs encoding GFP alone and GFP fused of wild type MSA1 (*GFP-MSA1w*) and mutated MSA1 (*GFP-MSA1m*) were transformed into *Arabidopsis* under the control of the CaMV 35S promoter. The GFP-MSA1 fusion protein was specifically expressed in the nucleus as stained by DAPI. Scale bar, 10μm.

To determine the tissue expression pattern of *MSA1*, WT was transformed with a *MSA1* promoter-GUS construct. In T2 transgenic seedlings a strong GUS signal was observed in roots and leaves, along with a weak signal in hypocotyls (Fig 3A–3C). In plants grown under S-sufficiency, GUS staining was mainly observed in the root maturation zone (Fig 3A). However, GUS staining was detected throughout the roots of the plants grown under S-deficiency (Fig 3B), indicating the *MSA1* promoter was activated by S-deficiency. This is confirmed by qRT-PCR (Fig 3H). GUS staining was also observed in the inflorescence (Fig 3D), especially in the stigma and anther (Fig 3E), and in the young siliques (Fig 3F) but not in the mature seeds (Fig 3G).

To investigate the subcellular localization of MSA1, a *GFP-MSA1* fusion construct under the control of the cauliflower mosaic virus (CaMV) 35S promoter was stably expressed in WT. GFP fluorescence was detected exclusively in the nucleus, as stained by the nuclear specific dye DAPI, suggesting MSA1 localizes to the nucleus (Fig 3I). Furthermore, the mutated MSA1 from *msa1-1* had the same nuclear localization as the WT protein (Fig 3I). The nuclear localization of MSA1 was not affected by S-deficiency and MSA1 localized to the nucleus in both leaves and roots (S5A and S5B Fig).

### Nuclear localization is required for MSA1 function

**Fig 4:**
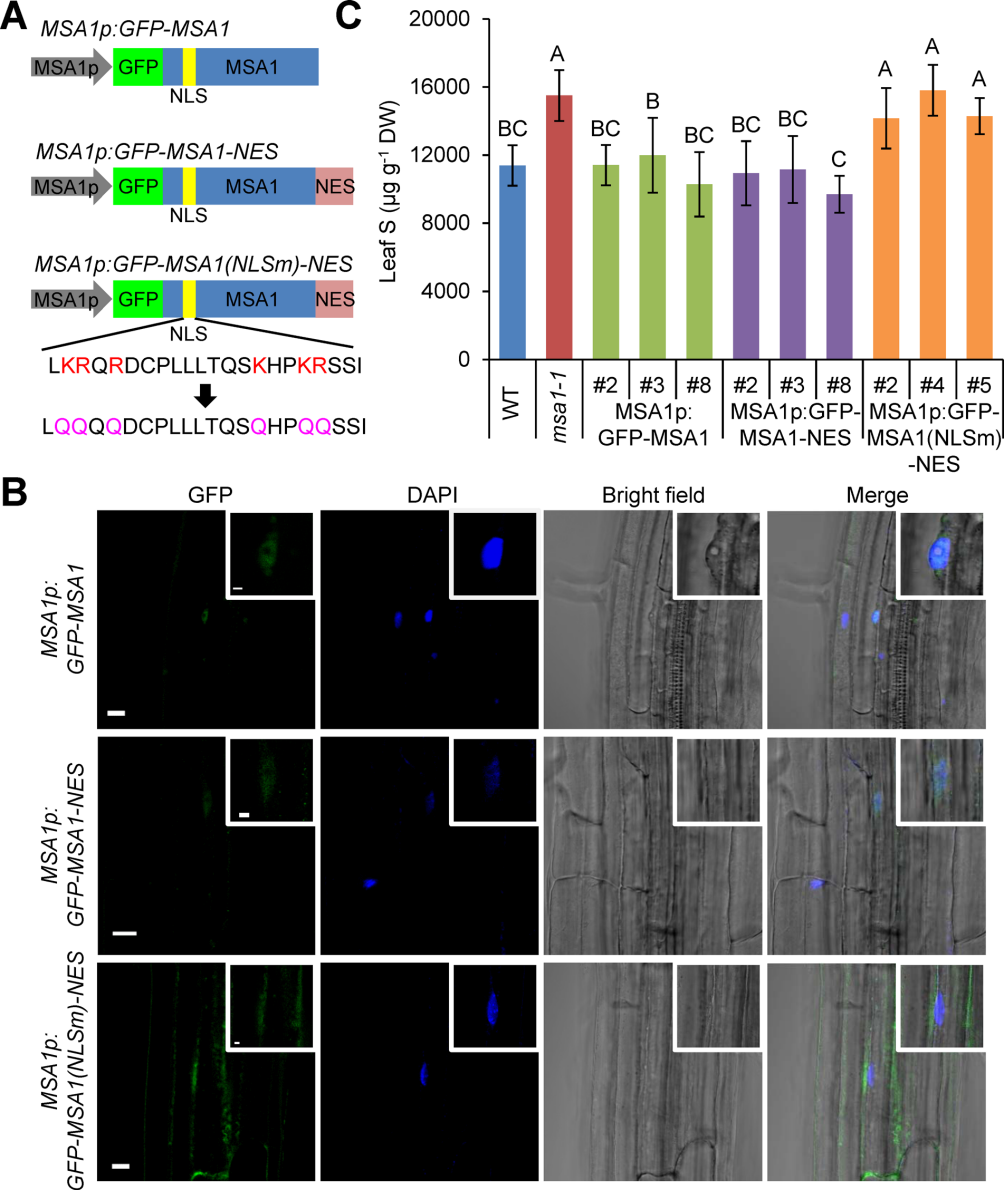
Localisation of MSA1 in cytosol could not suppress the high S phenotype of *msa1-1*. (A) Schematic diagram of the constructs used for transformation of the *msa1-1* mutant. MSA1p, *MSA1* promoter; NLS, nuclear localization signal; NES, nuclear export signal. (B) Subcellular localization of GFP-MSA1 in transgenic lines in the *msa1-1* mutant background transformed with the various constructs shown in (A). Insets show increased magnification of images showing the nucleus. Scale bar, 10μm and 2μm in the insets. (C) Total S in the leaves of five-week-old transgenic plants grown in soil. Three independent T2 lines are shown. Data presented as means ± SD (*n =* 9 to 12). Bars with different letters indicate significant differences (*P* ≤ 0.01, least significant difference test). DW, dry weight. ICP-MS data is accessible using the digital object identifier (DOI) 10.4231/T9PN93HT (see http://dx.doi.org/).

To explore whether nuclear localization is required for MSA1 function, we directed MSA1 into the cytosol by attaching a nuclear export signal (NES) at the C terminus of GFP-MSA1 and transforming the DNA construct into *msa1-1* with expression driven by the *MSA1* native promoter (Fig 4A). The NES is derived from the mammalian PKI protein and has been used to confer cytosolic localization of phytochrome B in *A*. *thaliana* [33, 34]. Similar to the *GFP-MSA1* control lines, the GFP signal was still found in the nucleus of *GFP-MSA1-NES* lines, suggesting that fusion of the NES to the C terminus of MSA1 is not sufficient to completely export MSA1 from the nucleus (Fig 4B). Not surprisingly, the total S in these lines was restored to WT levels (Fig 4C). MSA1 was predicted to have a putative bipartite nuclear localization signal (NLS) containing two clusters of lysine/arginine residues in the N terminus (S3A Fig). We mutated the native NLS of MSA1 by replacing both lysine and arginine with glutamines (Fig 4A). The basic amino acids lysine and arginine in an NLS are essential for the transport of nuclear localized proteins into the nucleus [35]. The *MSA1* construct with a mutated native NLS and fusion with NES at the C terminal was expressed in *msa1-1* from the *MSA1* native promoter. Nuclear localization of MSA1 was abolished in these transgenic lines, with the GFP signal being observed in the cytosol (Fig 4B). Total leaf S levels in these transgenic plants were the same as in *msa1-1*, demonstrating that MSA1 localized in the cytosol could not complement *msa1-1* (Fig 4C). These results indicated that nuclear localization is essential for MSA1 function in the regulation of S homeostasis.

### MSA1 lacks SHM activity *in vitro*

Given that *MSA1* had previously been annotated as SHM7 based on sequence homology without functional data [27], we tested whether MSA1 has SHM activity *in vitro* by expressing *MSA1* in *E*. *coli* and measuring SHM activity of purified protein with monoglutamylated THF as the substrate. We were able to detect the SHM activity of two well characterized SHM isoforms SHM1 and SHM2, with activities in the range previously reported [36]. However, no SHM activity was detected for MSA1 (S6A Fig). SHM enzymes exhibit different activity with monoglutamyl folate and polyglutamylated forms [37]. We have previously shown that SHM1, SHM2, SHM3 and SHM4 all exhibit SHM activity with either mono or polyglutamylated THF [38, 39]. However, we were unable to detect SHM activity of MSA1 using hexaglutamylated THF. Further, expression of *MSA1* in an *E*. *coli* loss-of-SHM function mutant [40] failed to rescue the glycine auxotrophy of this mutant (S6B Fig). Together these results strongly suggest that MSA1 is not a conventional SHM.

### Metabolite accumulation in *msa1-1*

**Fig 5:**
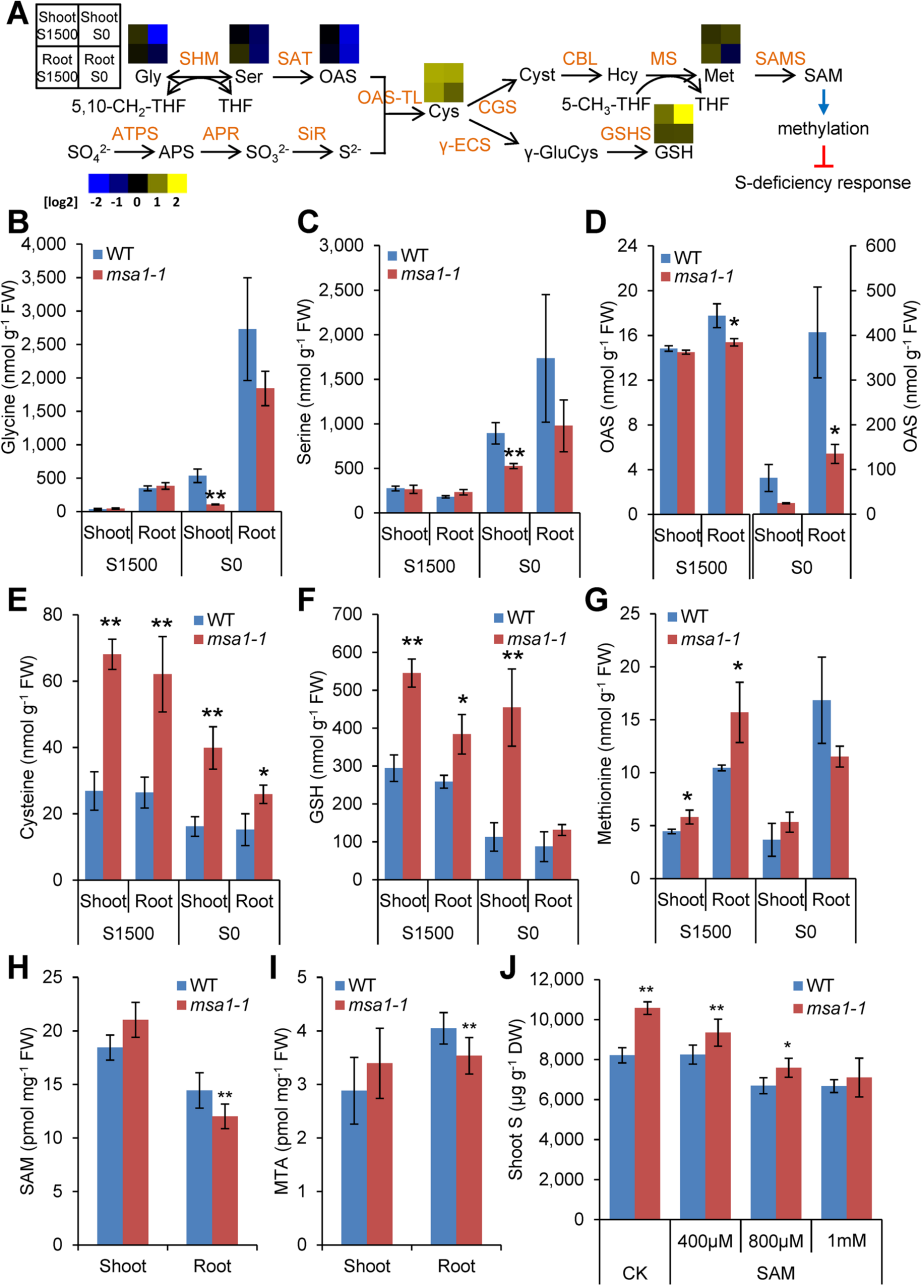
Metabolites quantification and supplementation. (A) Schematic representation of sulphur assimilation in *A*. *thaliana*. Colour squares above the metabolites represent the log2 value of the *msa1-1*/WT Col-0 ratio of the concentration of each metabolite. APR: APS reductase; APS: adenosine 5’-phosphosulfate; ATPS: ATP sulfurylase; CBL: cystathionine β-lyase; CGS: cystathionine γ-synthase; Cyst: cystathionine; γ-ECS: γ-glutamylcysteine synthetase; γ-GluCys: γ-glutamylcysteine; GSHS: glutathione synthetase; Hcy: homocysteine; MS: methionine synthase; OAS: O-acetylserine; OAS-TL: OAS(thiol)lyase; SAT: serine acetyltransferase; SAMS, S-adenosylmethionine synthetase; SiR: sulphite reductase; SHM: serine hydroxymethyltransferase. (B-G) Measurement of sulphur-related metabolites. Plants were grown on agar solidified MGRL media under S sufficient (S1500) or S deficient (S0) conditions. Metabolites were extracted from shoots and roots and quantified by HPLC. Data are presented as means ± SD (*n* = 3). *, *P* ≤ 0.05; **, *P* ≤ 0.01, Student’s *t* test. (H-I) The concentrations of SAM and MTA in the shoots and roots of WT Col-0 and *msa1-1* grown under S sufficient condition. (J) Total S in the shoots of WT Col-0 and *msa1-1* grown under S sufficient condition without (CK) or with SAM added to the growth medium. Data in (B-J) are presented as means ± SD (*n* = 3 in (B-G), *n* = 5 in (H-I), and *n* = 6 in (J)). * and ** in (B-J) indicate values significantly different between WT Col-0 and *msa1-1* mutant at *P* ≤ 0.05 and *P* ≤ 0.01, respectively (Student’s *t* test). DW, dry weight. CK, control.

To probe the function of MSA1 in S homeostasis, we determined the concentrations of S related metabolites in *msa1-1* grown under S sufficient and deficient conditions (Fig 5A). Under S-sufficiency, we observed no significant changes in the concentration of Ser and Gly in either shoots or roots of *msa1-1* (Fig 5B and 5C). The fact that Gly was not accumulated to high levels in shoots of *msa1-1* suggests that unlike the *shm1* knock-out mutant [41], *msa1-1* is not a photorespiratory mutant. OAS concentration was also not affected in shoots of *msa1-1* compared to WT but significantly decreased in roots under S-deficiency (Fig 5D). The *msa1-1* mutant accumulated higher levels of total S, as well as sulphate and sulphite, in shoots (Fig 1B–1D). We further showed that the S-containing amino acids Cys and Met and the Cys-containing tripeptide glutathione (GSH) were also elevated in both shoots and roots of *msa1-1* compared to WT (Fig 5E–5G). Under S-deficiency, the *msa1-1* mutant accumulated higher Cys in both shoots and roots but only high levels of GSH in shoots, and no significant difference of Met in either shoots or roots compared to WT was observed (Fig 5E–5G). These results indicated that not only the accumulation of sulphate but also S assimilation is enhanced in *msa1-1*. To summarize, *msa1-1* accumulates higher levels of Cys, Met and GSH. However, the *msa1-1* mutant generally maintained the same level of Gly, Ser and OAS under S-sufficiency as the WT but had lower levels under S-deficiency (Fig 5A). These results suggest that MSA1 is likely not a SHM involved in the conversion of Gly to Ser for the biosynthesis of Cys. However, under S-deficiency more Gly, Ser and OAS were used for the biosynthesis of Cys in *msa1-1*, consistent with the enhancement of S assimilation in *msa1-1*.

### Supplementation of SAM suppresses the high S phenotype of *msa1-1*

As Met is the precursor of the methyl group donor SAM, we further determined the concentration of SAM. We observed that the level of SAM, as well as 5'-methylthioadenosine (MTA), an intermediate of the endogenous Yang-cycle that recycles SAM after transfer of the methyl-group for synthesis of nicotinamide, polyamines or ethylene [42], were significantly lower in roots of *msa1-1* compared to WT (Fig 5H and 5I). To investigate whether a shortage of SAM might drive the high S phenotype of *msa1-1*, we performed a supplementation experiment. External supplementation with SAM in the growth medium completely suppressed the high S phenotype of *msa1-1* (Fig 5J). These results suggested that loss of function of *MSA1* results in a shortage of SAM leading to the high S phenotype of *msa1-1*.

### Effects of *MSA1* mutation on genome-wide DNA methylation

**Fig 6:**
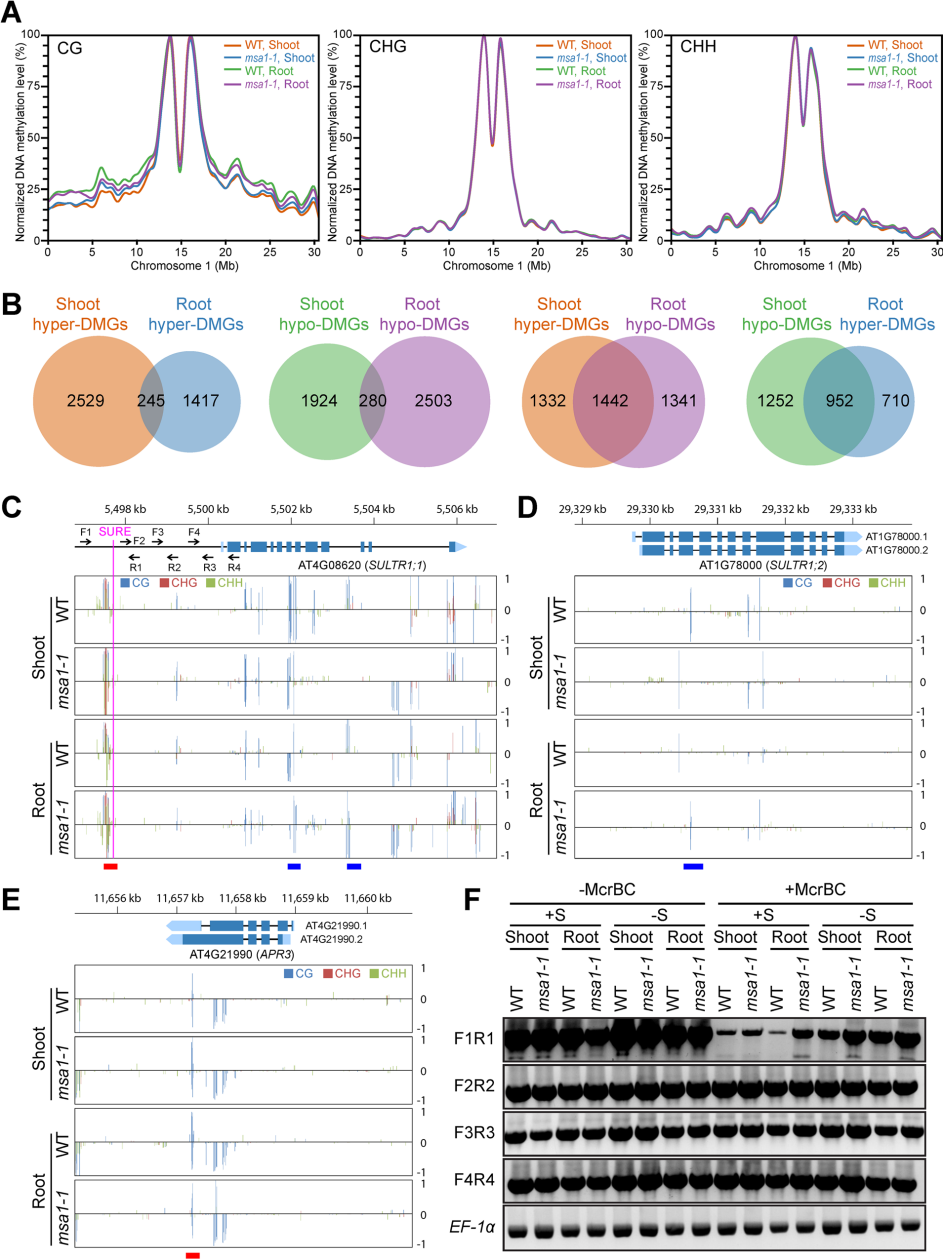
Effects of *MSA1* mutation on genome-wide DNA methylation. (A) Normalized DNA methylation level on CG, CHG and CHH contexts in the shoot and root of WT and *msa1-1* on chromosome 1. DNA methylation level was calculated as the density of methylated C in each 100 kb window, and the highest density window in each contexts was designated as 100%. See S7 Fig for other chromosomes. (B) Numbers of overlapping DMRs between the shoot and root of WT and *msa1-1*. (C-E) DNA methylation profile of *SULTR1;1* (C), *SULTR1;2* (D) and *APR3* (E) in the shoot and root of WT and *msa1-1*. DNA methylation levels are indicated by the height of vertical lines. The positive and negative values represent the methylation level in sense and antisense strand, respectively. The blue and red lines at the bottom indicated the location of the shoot and root DMRs, respectively. The vertical magenta line in (C) shows the location of the SURE element and arrows indicate the location of primers used for the chop-PCR in (F). (F) Determination of DNA methylation level in the promoter of *SULTR1;1* by chop-PCR in shoots and roots of WT and *msa1-1* plants grown on agar solidified MGRL media under S sufficient (1500 μM sulphate; +S) or S deficient (no added sulphate; -S) conditions. Genomic DNA was digested without or with McrBC, an endonuclease which only cleaves DNA containing methylcytosine residues, followed by PCR using the primers shown in (C).

Reduction of SAM levels by inhibition of folate biosynthesis has been shown to reduce global DNA methylation in *A*. *thaliana* [43]. Given that the concentration of SAM is reduced in roots of *msa1-1* (Fig 5J), we performed whole-genome bisulfite sequencing of WT and *msa1-1* to determine whether mutation of *MSA1* affects global DNA methylation. We achieved an average sequencing depth of 28 times, with more than 92% of cytosines in the nuclear genome covered, indicating the high-quality of our sequencing data (S1 Table). The overall cytosine methylation was lower in roots of *msa1-1* compared to the WT (Table 1). However, no reduction in DNA methylation was observed in *msa1-1* shoots (Table 1), and this may be due to the fact that SAM concentration is only reduced in roots but not in shoots of *msa1-1* (Fig 5J). Reduced DNA methylation in roots of *msa1-1* was found to be due to reduced cytosine methylation in both genes and transposon elements (TE) (S2 Table). Normalization of methylation level in 100 kb windows revealed that the overall difference in methylation between WT and *msa1-1* was mainly at cytosines in the CG sequence context and not in the CHG or CHH contexts (Figs 6A and S7). Using a sliding-windows approach, we identified 3,646 and 3,421 differentially methylated regions (DMRs) in the shoot and root between WT and *msa1-1*, respectively (S3 and S4 Tables). We defined genes with significant differential methylation (adjusted *p*-value < 0.05) in the gene body, and 2 kb upstream and 2 kb downstream as differentially methylated genes (DMGs). In total, 4,977 and 4,444 DMGs were identified in the shoot and root of *msa1-1*, respectively (S5 Table). Most of DMGs (> 92.7%) were differentially methylated on either the gene body or 2 kb upstream or downstream (S8 Fig). Among DMGs in the shoot, 2,773 are hyper-DMRs and 2,204 are hypo-DMRs showing significantly increased or decreased methylation, respectively. Among root DMGs, 1,662 are hyper-DMRs and 2,782 are hypo-DMRs. Comparison of the overlapping DMGs showed that only 14.7% hyper-DMGs in roots are hyper-methylated in shoots and 10.1% hypo-DMGs in roots are hypo-methylated in shoots. However, 51.8% hypo-DMGs in roots are hyper-methylated in shoots and 57.3% hyper-DMGs in roots are hypo-methylated in shoots (Fig 6B). Gene ontology enrichment analysis of DMGs revealed the enrichment of genes involved in various biological processes, especially in nucleotide binding (S6 Table).

**Table 1:** Cytosine methylation levels at CG, CHG, and CHH and total cytosine sites in WT and *msa1-1*.

*Sample*	CG	CHG	CHH	Total
*WT*, *Shoot*	28.06%	9.87%	3.05%	8.45%
*msa1-1*, *Shoot*	29.53%	10.62%	3.38%	9.09%
*WT*, *Root*	32.91%	12.42%	3.59%	10.29%
*msa1-1*, *Root*	29.42%	10.91%	3.09%	8.90%

To determine whether genes involved in S homeostasis are differently methylated in *msa1-1*, we searched DMRs for genes that have previously been shown to be responsive to S starvation, and also included glucosinolate and anthocyanin metabolisms genes which are involved in S homeostasis [44]. We identified four genes involved in glucosinolate and anthocyanin metabolisms and 15 S responsive genes that were differentially methylated in *msa1-1*, including two high-affinity sulphate transporter genes *SULTR1;1* and *SULTR1;2*, and genes encoding 5'-adenylylsulfate reductase (APR3) and ATP sulphurylase (APS4) (S7 Table and Fig 6C–6E). We found that the flanking sequence of the S responsive element (SURE) in the promoter of *SULTR1;1*, which is essential for the S deficiency response [13], was hypo-methylated in *msa1-1* roots (Fig 6C and S8 Table). Using chop-PCR, we confirmed that the SURE flanking sequence of *SULTR1;1* is hypo-methylated in *msa1-1* roots (Fig 6F). Significantly, using chop-PCR we also show that in WT this SURE flanking sequence appears to be hyper-methylated in S sufficient conditions and hypo-methylated in S deficient conditions (Fig 6F).

### Expression of S response genes is upregulated in *msa1-1*

**Fig 7:**
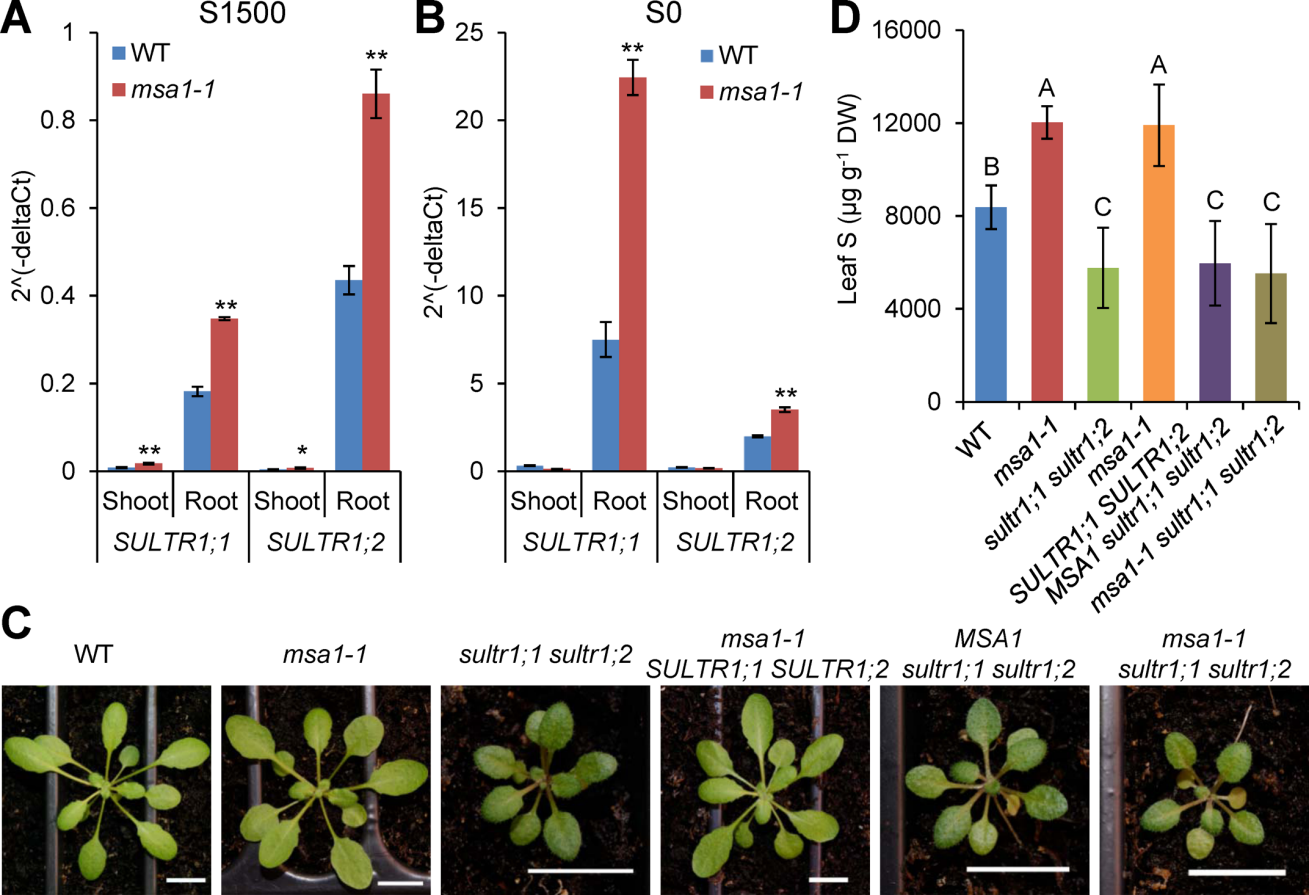
High leaf S phenotype of *msa1-1* is dependent on two high-affinity sulphate transporters SULTR1;1 and SULTR1;2. (A, B) Expression of *SULTR1;1* and *SULTR1;2* in the *msa1-1* mutant. Quantitative RT-PCR analysis of *SULTR1;1* and *SULTR1;2* in the shoot and root of WT Col-0 and *msa1-1*. Plants were grown on agar solidified MGRL media for two weeks with sufficient sulphate (1500 μM; S1500) (A) or without added sulphate (S0) (B). Expression level was normalized to the internal control gene *UBQ10*, and presented as 2^(-deltaCt) with means ± SD (*n =* 3). * and ** represent significant differences between the WT and mutant at *P* ≤ 0.05 and *P* ≤ 0.01, respectively (Student’s *t* test). (C) Phenotype of five-week-old *msa1-1 sultr1;1 sultr1;2* triple mutant and control lines. Pictures were taken before harvesting for ICP-MS analysis. Scale bars in all images represent 1 cm. (D) Total S in the leaves of five-week-old *msa1-1 sultr1;1 sultr1;2* triple mutant and control lines. Data are presented as means ± SD (*n =* 11 or 12). Bars with different letters indicate significant differences (*P* ≤ 0.01, least significant difference test). DW, dry weight.

To better understand the connection between *MSA1* function, DNA methylation and the elevated accumulation of total S in leaves of *msa1-1*, we investigated the expression level of genes involved in S homeostasis. Quantitative RT-PCR revealed that expression of *SULTR1;1*, *SULTR1;2*, and *SULTR4;2* genes encoding sulphate transporters involved in root uptake and translocation of sulphate was higher in roots of *msa1-1* compared to WT (S9 Fig). Further, transcription of genes encoding the APS reductases APR1, APR2, and APR3, which are required for sulphate reductive assimilation, were also increased in roots of *msa1-1* compared to WT (S9 Fig). The increased expression of *SULTR1;1* and *SULTR1;2* in the roots of *msa1-1* was confirmed in plants grown on agar-solidified media (Fig 7A and 7B). These observations support the conclusion that the enhanced sulphur uptake and assimilation of *msa1-1* is driven by constitutive induction of S-deficiency response genes. To directly test if the high leaf S phenotype of *msa1-1* is dependent on the high-affinity sulphate transporters SULTR1;1 and SULTR1;2, we generated an *msa1-1 sultr1;1 sultr1;2* triple mutant by crossing *msa1-1* with the *sultr1;1 sultr1;2* double knockout mutant in the Wassilewskija (Ws) background [45] (Figs 7C and S10). To exclude the possibility that the different genetic backgrounds of Col-0 and Ws may affect the S phenotype, we selected one *msa1-1 SULTR1;1 SULTR1;2* line which has the *msa1-1* mutant allele and *SULTR1;1* and *SULTR1;2* WT alleles, and one *MSA1 sultr1;1 sultr1;2* line with the *MSA1* WT allele in a *sultr1;1 sultr1;2* double knockout background (Figs 7C and S10). The leaf S concentration of the *sultr1;1 sultr1;2* double mutant was significantly lower than WT (Fig 7D), consistent with its low sulphate uptake rate [45, 46]. There was no significant difference in leaf S concentration between *msa1-1* and the *msa1-1 SULTR1;1 SULTR1;2* line, or between *sultr1;1 sultr1;2* double knockout and the *MSA1 sultr1;1 sultr1;2* line, suggesting that the differences in genetic backgrounds between Col-0 and Ws had no effect on the leaf S phenotype (Fig 7D). The leaf S concentration of the *msa1-1 sultr1;1 sultr1;2* triple mutant was similar to the *sultr1;1 sultr1;2* double mutant and the *MSA1 sultr1;1 sultr1;2* line indicating that the *MSA1* mutation acts through elevated expression of *SULTR1;1* and *SULTR1;2* to enhance S accumulation (Fig 7D).

## Discussion

The uptake, assimilation and metabolism of S in plants has been well explored. However, our understanding of the regulation of S homeostasis remains much more limited. In this study, we present evidence supporting the function of the nuclear-localized MSA1 in controlling S homeostasis in *A*. *thaliana*. Loss of function of *MSA1* reduces SAM levels, alters genome-wide DNA methylation levels, and leads to a constitutive S deficiency response (Fig 5A). MSA1 shows a high level of sequence homology to well characterised SHM enzymes, and recent studies have shown that yeast SHM2, as part of a larger complex with other proteins, is involved in the biosynthesis of SAM in the nucleus for histone methylation [47]. We have established that MSA1 localizes to the nucleus, and that this nuclear localization is essential for MSA1 function (Figs 3I and 4A–4C). We therefore propose that MSA1 functions in the production of a nuclear pool of SAM, though the existence of such a pool of SAM remains to be directly tested. However, our hypothesis is supported by the known nuclear localization of various enzymes involved in SAM biosynthesis in *A*. *thaliana*, including the SAM synthetases AtSAMS1, AtSAMS2 and AtSAMS3 [48–50], and enzymes involved in the recycling of the by-products of SAM-dependent transmethylation, including the SAH hydrolases SAHH1 and SAHH2, and adenosine kinase ADK1 [51].

Blockage of SAM biosynthesis by inhibition of folate biosynthesis using sulfamethazine has previously been shown to reduce global DNA methylation [43]. Here, we show that the overall level of DNA methylation is reduced in *msa1-1* roots (Table 1), which is consistent with the reduced SAM concentration in this tissue (Fig 5J). Sulphate uptake and assimilation are repressed in normal S supply and de-repressed during S deficiency [4, 5]. In *msa1-1* we identified several differentially methylated genes which are known to be responsive to S deficiency (S7 Table), including two high-affinity sulphate transporter genes *SULTR1;1* and *SULTR1;2*, and the S assimilation gene *APR3* (Fig 6C–6E) which are hypo-methylated in *msa1-1* compared to WT. Furthermore, *msa1-1* shows a strong constitutive S-deficiency response, including increased expression of *SULTR1;1*, *SULTR1;2* and *APR3* (Figs 7A, 7B and S9). These results suggest that de-repression of S responsive genes at ample S supply in *msa1-1* is likely caused by their differential methylation, and it is this de-repression that leads to the strong constitutive S-deficiency response in *msa1-1*. This is exemplified by the sulphur responsive element SURE upstream of *SULTR1;1*. In WT the flanking sequence of this element is hyper-methylated in S sufficient condition but hypo-methylated in S deficient condition (Fig 6F). Whereas, we observed that the flanking sequence of this SURE element is hypo-methylated in *msa1-1* even under S sufficient conditions (Fig 6F). This suggests that the constitutive elevation of expression of *SULTR1;1* in *msa1-1* is due to the hypo-methylation of the SURE element in its promoter. Similarly, hypo- and hyper-methylation in the vicinity of *cis*-acting elements known to regulate expression of phosphate–responsive genes have been shown to correlate with increased or decreased expression of low-phosphate responsive genes [52]. Furthermore, *SULTR1;3* is hypo-methylated and its expression is upregulated under phosphate starvation [18], indicating another example of regulation of *SULTR* gene expression by DNA methylation. The concurrence of hypo-methylation of *SULTR1;1* as well as *SULTR1;3* and their upregulated expressions suggests the existence of a potentially common mechanism in the regulation of *SULTR* transporter gene expression through altered DNA methylation under nutrient deficiency.

Previous studies have shown that expression of *MSA1* is regulated by *SLIM1* [7]. *MSA1* expression is significantly elevated by S-deficiency (Fig 3H). Meanwhile, the expression of *SULTR1;1* and *SULTR1;2* is also induced by S-deficiency (Fig 7A and 7B). This suggests that the induction of *SULTR1;1* and *SULTR1;2* under S-deficiency by demethylation is not controlled by the upregulation of *MSA1* which would be expected to enhance DNA methylation by increasing SAM supply. One possible function of MSA1 under S-deficiency could be in prioritising SAM biosynthesis in the nucleus to maintain overall DNA methylation, and this is supported by our observation of an overall decrease in DNA methylation in roots of *msa1-1*. A second possibility is that the upregulation of *MSA1* under S-deficiency is to specifically suppress, by methylation, genes down-regulated during the S-deficiency response. This possibility is supported by the fact that the biosynthesis of glucosinolates, a group of S-rich secondary metabolites, is inhibited during S-deficiency, and many genes involved in their biosynthesis are strongly down-regulated, such as genes encoding a branched-chain amino acid aminotransferase, methylthioalkylmalate synthases, and cytochrome P450s [7, 53]. Interestingly, the branched-chain amino acid aminotransferase genes *BCAT3* and *BCAT4*, and the cytochrome P450 gene *CAP79B2* are differentially methylated between WT and *msa1-1* (S7 Table), supporting this hypothesis. However, further studies are required to test the idea that methylation suppresses expression of genes involved in S consumption. It is also possible that *MSA1* and *SULTR1;1* as well as *SULTR1;2* are induced in different cell types under S-deficiency. Such cell-type-specific induction by S-deficiency has been observed for miR395 and *SULTR2;1*. The induction of miR395 by S-deficiency is restricted to the phloem companion cells in roots, which fails to digest the miRNA target *SULTR2;1* expressed in xylem parenchyma and pericycle cells leaving the *SULTR2;1* mRNA intact [8].

SAM is also the methyl donor for RNA methylation. It is possible that the decreased SAM pool in *msa1-1* might reduce RNA methylation of sulphur deficiency responsive genes and thus affect their expression. Methylation at the N^6^ of adenosine (m^6^A) on messenger RNA (mRNA) has been shown to be correlated with mRNA abundance in *A*. *thaliana* [54, 55]. It is also possible that loss of function of *MSA1* affects histone methylation, and that the increased expression of S responsive genes in *msa1-1* may be also due to differential methylation of histones. Mutation of the folylpolyglutamate synthetase *FPGS1* that disrupts folate and SAM metabolism has been shown to reduce global DNA and H3K9 dimethylation in *A*. *thaliana* [56], and deletion of *SHM2* in yeast was observed to reduce H3K4 methylation [47]. Consistent with this, several S deficiency response genes in *A*. *thaliana* have been identified as targets of the trimethylated histone 3 H3K27me3 [57]. Therefore, we hypothesise that MSA1 is involved in maintaining an adequate pool of SAM in the nucleus, though the mechanism remains unclear. This pool of SAM is required for DNA methylation, including that underpinning the epigenetic regulation of S homeostasis.

## Materials and Methods

### Plant materials and growth conditions

The T-DNA insertion mutants for At1g36370 (*MSA1*, SALK_044268 and SALK_118251) and for At1g23935 (SALK_069606) were obtained from the Arabidopsis Biological Resource Center (ABRC, http://www.arabidopsis.org/abrc/). The *sultr1;1 sultr1;2* double mutant was kindly provided by Dr. Hideki Takahashi. The *msa1-1 sultr1;1 sultr1;2* triple mutant was generated by crossing *sultr1;1 sultr1;2* double mutant with *msa1-1* and the homozygous triple mutant was selected from the F2 population using the primers listed in S9 Table. *A*. *thaliana* plants for ICP–MS analysis were grown as described previously [20]. Briefly, seeds were germinated on moist soil (Pro-Mix (Premier Horticulture) or Bulrush multipurpose compost) in a 20-row tray. After stratification at 4°C for 3 days, plants were grown in a climate-controlled room at 19–22°C with photoperiod of 10 h light (100 ± 10 μmol m^-2^ s^-1^)/14 h dark and humidity 60%. Plants were bottom-watered at regular intervals with modified 0.25× Hoagland solution [20]. For plants grown in axenic conditions, surface sterilized seeds were vertically grown on MGRL agar media [58] with 1% UltraPure sucrose (Sigma) at 22°C with photoperiod of 16 h light (100 μmol m^-2^ s^-1^)/8 h dark. For preparation of agar medium, agar (Sigma, type A) was washed three times with 5 liters of deionized water and vacuum filtrated to dry. Sulphur deficiency agar medium (S0) was prepared by replacement of MgSO_4_ with MgCl_2_.

### Tissue elemental analysis

The determination of tissue elemental concentration was performed as described previously [20]. For plants grown in soil, 1 to 2 leaves of five-week-old plants were harvested for analysis. For plants grown on agar plates, shoots or roots of 4 to 5 two-week-old plants were combined as one sample separately for analysis. Elemental analysis for Li, B, Na, Mg, P, S, K, Ca, Mn, Fe, Co, Ni, Cu, Zn, As, Se, Rb, Sr, Mo and Cd was performed with an inductively couple plasma mass spectrometer (Elan DRC II, PerkinElmer; or NexION 300D, PerkinElmer). For the plants grown in soil, data for elements are available in the iHUB (www.ionomicshub.org).

### Bulk segregant analysis (BSA) and re-sequencing of *msa1-1*

SNP-tilling array-based bulk segregant analysis was performed as previously described [59]. Briefly, 40 F2 plants with high leaf S or normal S compared to Col-0 WT, from a cross between *msa1-1* and L*er*-0, were pooled separately. Genomic DNA was extracted from the two pools using a DNeasy Plant Maxi Kit (Qiagen) and labelled using a BioPrime DNA labelling system (Invitrogen). The labelled pooled DNA was separately hybridized to the Affymetrix 250K SNP-tilling array ATSNPTILE1. The single nucleotide polymorphisms (SNPs) previously established between L*er*-0 and Col-0 were used as genetic markers. The allele frequency difference between the two pools was assessed using the R scripts as described by Becker et al. [59]. For re-sequencing of the *msa1-1* mutant, DNA was extracted using a DNeasy Plant Mini Kit (Qiagen) and sequenced on an ABI SOLiD (Applied Biosystems). The sequencing was performed according to the manufacturer’s instructions. The short reads were aligned to the Col-0 reference genome and the SNPs were identified in the BSA confidence interval.

### Transgenic complementation

To create a complementation construct, the genomic DNA fragment of *MSA1* was PCR amplified from Col-0 genomic DNA using the primers as listed in S9 Table and then ligated into pCR™2.1-TOPO vector (Invitrogen) for sequencing. The 6,116-bp fragment (containing the full-length *MSA1* genomic sequence, the 2,925-bp sequence before the ATG and the 854-bp sequence after the TAG) was released with *Eco*RI and *Pst*I and ligated into the binary vector pCAMBIA1301. The resulting plasmid was transformed into *Agrobacterium tumefaciens* strain GV3101 and introduced in *msa1-1* using the floral dip method [60]. Transgenic plants were screened on half-strength MS medium containing 50 mg ml^-1^ hygromycin.

### Subcellular localization and promoter-GUS expression pattern of MSA1

To investigate the subcellular location of MSA1, the full-length coding sequence of *MSA1* was amplified from cDNA of Col-0 WT or *msa1-1* and ligated into pCR™2.1-TOPO vector (Invitrogen) for sequencing. The coding sequence of MSA1 after sequencing was released from the vector using *Spe*I and *Sma*I and ligated into p1301GFP vector [61] to form the construct *35S*:*GFP-MSA1*. The resulting plasmid was transformed into *A*. *tumefaciens* strain GV3101 and introduced into *A*. *thaliana* using the floral dip method [60]. Transgenic plants were screened on MGRL media containing 50 mg ml^-1^ hygromycin. Five-day-old transgenic plants grown on MGRL media with or without sulphate were examined using a confocal laser-scanning microscope (Carl Zeiss LSM700). To visualize the nuclei plants were incubated with 1 μg mL^-1^ of 4’,6-diamidino-2-phenylindole (DAPI; Molecular Probes) for 5 min and then washed 5 times with PBS buffer. GFP were excited using an argon laser at 488 nm; the emission was collected between 505 and 530 nm. DAPI were excited with a UV laser at 395 nm and the emission was recorded between 440 and 470 nm. For analysis of *MSA1* tissue expression pattern, the 2,628-bp promoter sequence of *MSA1* was PCR amplified and subcloned into vector p1300-GN [61] to create a fusion of the *MSA1* promoter with the β-glucuronidase (GUS) reporter gene (*MSA1p*:*GUS*). Transgenic plants were generated as described above. GUS histochemical staining was performed as described previously [61]. The primer sequences used are listed in S9 Table.

### Localization of MSA1 to the cytosol

To generate the *MSA1p*:*GFP-MSA1* construct, the *GFP*-*MSA1* sequence was released from the *35S*:*GFP-MSA1* construct using *Sal*I and *EcoR*I and ligated into pCAMBIA1301 to form *p1301-GFP-MSA1*. The *MSA1* promoter sequence was released from the *MSA1* promoter GUS construct *MSA1p*:*GUS* using *Pst*I and *Sma*I and inserted into the *Pst*I and blunted *Sal*I sites of *p1301-GFP-MSA1* to form the *MSA1p*:*GFP-MSA1* construct. For construction of the nuclear export signal (NES) *MSA1* fusion plasmid (*MSA1p*:*GFP-MSA1-NES*), the *MSA1-NES* sequence was amplified by PCR with the primers containing the coding sequence of NES from the mammalian PKI protein [33, 34]. The sequenced *GFP-MSA1-NES* fragment and the *MSA1* promoter fragment were ligated into the pCAMBIA1301 vector as above to generate the *MSA1p*:*GFP-MSA1-NES* plasmid. The NLS of MSA1 was predicted by cNLS Mapper [62]. The mutagenesis of the MSA1 NLS was carried out by an overlap extension. The same strategy was used to clone the *GFP-MSA1(NLSm)-NES* fragment and *MSA1* promoter fragment into the pCAMBIA1301 vector as above to generate the *MSA1p*:*GFP-MSA1(NLSm)-NES* plasmid. All constructs were confirmed by sequencing and transformed into *msa1-1*. The transformation, screening and ICP-MS analysis was done as described above. The primer sequences used are listed in S9 Table.

### RNA extraction, cDNA synthesis and quantitative real-time PCR

Total RNA was extracted using a TRIzol Plus RNA Purification kit (Invitrogen, Life Technologies), and then treated with RNase-Free DNase I (Thermo Scientific) to remove potential genomic DNA contamination. The cDNA synthesis was carried out using a SuperScript VILO cDNA Synthesis Kit (Invitrogen, Life Technologies). Quantitative real-time PCR was performed on an ABI StepOnePlus Real-Time PCR System (Applied Biosystems) using SYBR Green PCR Master Mix (Applied Biosystems) or Maxima SYBR Green qPCR Master Mixes (Thermo Scientific). The cycling conditions were set according to the instrument’s instructions. The CT values were normalized to the corresponding *UBQ10* gene (At4g05320). The primer sequences used are listed in S9 Table.

### Metabolite quantification

*Arabidopsis thaliana* plants were vertically grown on either normal (1500 μM sulphate, S1500) or sulphate deficiency (S0) agar solidified medium for 2 weeks as described above. Plant tissues were harvested and weighed in 1.5 mL centrifuge tubes, immediately frozen in liquid nitrogen, and stored at -80°C for further analysis. For analysis of Ser, Gly and Met, frozen plant material was ground with a pestle to a fine powder in a 1.5 mL centrifuge tube and free amino acids extracted as described previously [63]. Briefly, finely powdered tissues were extracted twice with water:chloroform:methanol (3:5:12, v/v) in a ratio of 3 μL for each milligram of tissue. γ-aminobutyric acid was added as an internal standard. After brief centrifugation, the combined supernatant was mixed with chloroform and water and re-centrifuged. The upper water-methanol phase was collected, dried using a Speedvac (Thermo Scientific), and dissolved in water. Amino acids were derivatized with 6-aminoquinolyl-*N*-hydroxysuccinimidyl carbamate using an AccQ·Fluor Reagent Kit (Waters) according to the manufacturer’s instructions. Amino Acid Standard H (Thermo Scientific) was used for establishing standard curves. HPLC was performed using an Agilent 1100 series (Agilent Technologies) and separations were done on a 4.6 × 150 mm Alltima HP C18 column (Alltech Associates, PN:87679). The separation program and solvent system was prepared according to Goyer et al. [64]. Eluted amino acid-derivatives were detected using a G1321 model Agilent 1100 series with an excitation wavelength of 250 nm and an emission wavelength of 395 nm.

The extraction and derivatization of GSH and cysteine was performed as described previously [65]. HPLC was done as described above with the elution method described by Tsakraklides et al. [65]. The GSH and cysteine derivatives were detected with the fluorescence detector G1321 model (Agilent Technologies) with an excitation wavelength of 360 nm and an emission wavelength of 450 nm. OAS was quantified after derivatization with AccQ-Tag reagent (Waters) and HPLC separation [66]. Sulphate was determined by anion exchange HPLC method and sulphite was measured after derivatisation with monobromobimane and HPLC separation [67]. SAM and MTA were quantified as previously described [68].

### Heterologous expression and SHM activity

To clone *MSA1* for SHM activity assays using monoglutamylated THF as the substrate, the coding sequences were amplified from cDNA prepared from leaf mRNA of Col-0 (Nucleospin RNA Plant Kit, Macherey-Nagel; RevertAid H minus cDNA synthesis kit, MBI Fermentas) using the oligonucleotides listed in S9 Table. Excised cDNA fragments were ligated into the *EcoR*I-*Kpn*I site of the *E*. *coli* overexpression vector pHUE [69] as histidine-tagged ubiquitin fusions. Correctness of constructs was verified by sequencing. The overexpressed proteins were purified by Ni-affinity chromatography (Quiaexpress, Quiagen), and before assessing SHM activity, the fusions were cleaved with a recombinant histidine-tagged deubiquitylating protease Usp22_cc as described in Catanzariti et al. [69]. Adequate cleavage was confirmed by sodium dodecyl sulphate (SDS) polyacrylamide electrophoresis. Recombinant mature SHM1 and SHM2 were prepared as previously described [36] and used as positive controls. SHM activities of all three recombinant proteins and empty-vector controls were measured according to Taylor and Weissbach [70] using 3- [^14^C]-L-serine and monoglutamylated tetrahydrofolate as substrates as previously described in detail [71].

To clone *MSA1* for SHM activity assay using hexaglutamylated THF as the substrate, total RNA was isolated from the leaf of Col-0 using the RNeasy Plant Mini Kit (Qiagen, Valencia, CA), and the cDNA was reverse-transcribed using Superscript II reverse transcriptase (Invitrogen) and an oligo78 (dT) primer. The open reading frames were then amplified using Taq2000 polymerase (Stratagene, La Jolla, CA) using the primers listed in S9 Table. The generated PCR fragments were purified using a Wizard PCR column (Promega) and cloned into the pGEM-T Easy vector (Promega). The primers comprised gene-specific sequences flanked by vector-specific sequences needed for cloning into pET-30 Ek/LIC expression vectors (Novagen). The ORFs were re-amplified from the generated constructs using Pfu polymerase (Stratagene) and the same primers listed in S9 Table. The generated PCR fragments were purified using Wizard PCR columns, treated with T4 polymerase, and ligated into the pET-30 Ek/LIC vector following the manufacturer's protocol. All constructs were verified by sequencing. The pET-30 Ek/LIC constructs above were introduced into the Rosetta strain of *E*. *coli* (Novagen) to express the recombinant proteins. Bacteria were cultured at 37°C in LB medium containing 100 μg ml^-1^ kanamycin and 34 μg ml^-1^ chloramphenicol. When an optical density at 600nm of 0.6–1 was reached, isopropyl-D-thiogalactopyranoside (IPTG) was added to a final concentration of 1 mM, and the incubation continued overnight at 15°C. The induced bacteria were pelleted at 5,000 g for 15 min at 4°C. The collected bacteria were frozen in liquid N_2_ and stored at −80°C until use. The recombinant enzymes were purified from the bacterial lysate using an Äkta FPLC system equipped with 1-ml IMAC column (GE Healthcare) charged with Ni^2+^ according to the manufacturer’s protocol. All chromatography steps were performed at 4°C. A charged column was equilibrated with Binding Buffer (20 mM CHC [Ches-Hepes-citric acid], pH 7.5, 500 mM NaCl, 20 mM imidazole, 0.5 mM THP, 0.25 mM PLP, and 10% glycerol) before loading the clarified cell lysates. Unbound proteins were removed by washing with 15 column volumes of Binding Buffer, followed by elution of bound proteins by a linear gradient of Binding Buffer to Elution Buffer (20 mM CHC, pH 7.5, 500 mM NaCl, 20 mM imidazole, 0.5 mM THP, 0.25 mM PLP, and 10% glycerol) over 15 column volumes. Both recombinant proteins eluted at about 200 mM imidazole. Fractions containing the recombinant proteins were pooled and immediately desalted into Protein Storage Buffer (50 mM CHC, pH 7.5, 1 mM THP, 0.25 mM PLP, and 10% glycerol) using PD-10 desalting columns (GE Healthcare). The desalted samples were aliquoted and stored at -80°C until use. The enzymes were assayed at 30°C for 20 min with 10 μM hexaglutamylated THF and 5 mM serine as substrates using an HPLC-based fluorometric assay as described before [72]. The resolution limit of the assay was < 1 nmol min^-1^ mg^-1^ protein. No product peaks were visible in any of the blanks or assays.

### *E*. *coli* complementation experiment

The complementation of an *E*. *coli* glycine auxotroph (GS245(DE3)pLysS; *shmt*^-^) was performed as previously described with modifications [40, 73]. The coding sequences of *MSA1* were commercially synthesized to optimize the codon usage for expression in *E*. *coli*, and then ligated into the *EcoR*I-*Sal*I site of pET28a and the *Sac*I-*Xho*I site of pET32a, respectively. The coding sequence of *E*. *coli SHMT* gene was PCR amplified from genomic DNA of *E*. *coli* strain Top10 using the primer sequences listed in S9 Table and ligate to the *EcoR*I-*Sal*I site of pET32a. After confirmation by sequencing the correct constructs were transformed into GS245(DE3)pLysS. Three independent clones of each GS245(DE3)pLysS strain carrying different plasmids were grown in Lysogeny broth (LB) medium overnight at 37°C. Cells were collected by centrifugation and washed twice with M9 minimal medium (1× M9 salts (Sigma), 0.4% w/v glucose, 50 μg ml^-1^ phenylalanine, 10 μg ml^-1^ thiamine, 2 mM MgSO_4_, 0.1 mM CaCl_2_, 5 μM FeSO_4_, 3 nM (NH_4_)_6_Mo_7_O_24_, 400 nM H_3_BO_3_, 30 nM CoCl_2_, 10 nM CuSO_4_, 80 nM MnCl_2_, and 10 nM ZnSO_4_). Cells were re-suspended and diluted in M9 minimal medium to an equal optical density at 630nm of 0.5. Ten micro litres of cells were transferred to 1 mL of M9 minimal medium with required antibiotics in a 96-well 2 mL deep plate. Different concentrations of IPTG were added for induction. 96-well plates were placed in an orbital shaker and incubated at 37°C and 350 rpm. The OD630 value was measured after 24 h incubation using a plate reader.

### Whole-genome bisulfite sequencing and data analysis

Plants used for whole-genome bisulfite sequencing were grown on MGRL media for two weeks. Genomic DNA was extracted from shoots and roots of WT or *msa1-1* using a DNeasy Plant Mini Kit (Qiagen). DNA samples were sent to Beijing Genomics Institute at Shenzhen (BGI, Shenzhen, China) for bisulfite treatment, library construction and high throughput sequencing. Bisulfite treatment was carried out by an EZ DNA Methylation-Gold kit (ZYMO Research). For data analysis, the adapter sequences were trimmed and low-quality reads (q < 20) were removed. Clean reads were mapped to the *A*. *thaliana* genome (TAIR10) using BSMAP aligner [74] with reads length*0.08 mismatches allowed. Only reads mapped to unique positions on the genome were retained for DNA methylation analysis. The DNA methylation level was calculated only on those cytosine sites with at least fourfold coverage. The methylation level for each cytosine was calculated by dividing the number of reads covering methylated cytosine by the total number of effective sequencing reads at that cytosine site. The conversion rates of cytosine were more than 99.57% as estimated the methylation rate of chloroplast genome (S1 Table).

A sliding-windows approach with five CGs (CHGs or CHHs) as a window at one C interval was used to identify the DMRs. DMRs were identified by comparing the methylation level at CG, CHG and CHH contexts separately. A Fisher’s exact test was used to determine the DMR between samples. *P*-values calculated from the test were then adjusted using the Benjamini-Hochberg method to control the false discovery rate (FDR). Windows with adjusted *P*-value ≤ 0.05, fold-change of methylation level between two samples > 2 and coverage > 0.6 was considered as a DMR. Overlapped windows was merged into DMRs and filtered to include only those with minimum 200 bp length. Genes with at least one DMR in the gene body, 2 kb upstream, or 2 kb downstream flanking regions were considered as differentially methylated genes (DMGs). Gene ontology (GO) enrichment analysis was performed on hyper- and hypo-DMGs of shoots and roots, and the overlapped DMGs using DAVID [75]. Only the annotation cluster with enrichment score > 2 were showed in S6 Table.

### Chop-PCR

WT and *msa1-1* plants were grown on MGRL media with 1500 μM (+S) or without (-S) sulphate for two weeks. The genomic DNA was extracted from shoots and roots using a DNeasy Plant Mini Kit (Qiagen). One microgram genomic DNA was mixed with restriction buffer and then split into 2 × 50 μL with either 2 μL McrBC enzyme (New England Biolabs) or 2 μL 2% (v/v) glycerol as nondigested control. After overnight digestion and deactivation, a standard PCR was performed using the primers to amplify the promoter sequence of *SULTR1;1*. *EF-1α* (AT5G60390) was used as a negative control. The primer sequences used for PCR are listed in S9 Table.

### Homology modelling and sequence alignment

Homology modelling was conducted using the web-based SWISS-MODEL platform (http://swissmodel.expasy.org) [76]. The crystal structure of homotetrameric rabbit cytosolic SHMT1 (PDB ID: 1ls3) [32] with bound ligands was used as a template and the model was built in a monomer manner. Given the low sequence similarity in the N- and C-termini of MSA1 with the template, the model was only built from residues 136 to 591 of MSA1. The model quality was evaluated using the structure assessment tools of the Swiss-Model workspace. Structures were viewed using the DeepView/Swiss-PdbViewer 4.1 (http://www.expasy.org/spdbv/) [77]. The MUTATE tool in DeepView/Swiss-PdbViewer 4.1 was used to mutate Ser186 to Phe. The clash scores the five rotamers generated from the rotamer library (Rotolib.aa) are 10, 10, 10, 12 and 22. S3B Fig showed the rotamer with a clash score of 12. Multiple sequence alignments of SHM proteins were conducted using BioEdit software with the ClustalW method.

## Supporting Information

S1 Fig The S concentration in WT and the *msa1-1* mutant grown under various concentration of sulphate. Plants were grown on MGRL agar media without sulphate (S0), with 15 μM (S15), 1500 μM (S1500) or 4500 μM (S4500) sulphate for two weeks. Total S in both shoots (A) and root (B) were determined. Data are presented as means ± SD (*n* = 6). Single and double asterisks indicate values significantly different between WT and *msa1-1* mutant at *P* ≤ 0.05 and *P* ≤ 0.01, respectively (Student’s *t* test). DW, dry weight.
(TIF)

S2 Fig The *msa1-1* mutant is a recessive mutant. (A, B) S and Se content in the leaves of WT Col-0, L*er*-0, *msa1-1* and *msa1-1* X L*er*-0 F1 plants. Data are presented as means ± SD (*n =* 12). Columns with different letters indicate significant difference (*P* ≤ 0.01, least significant difference test). DW, dry weight. (C, D) The frequency distribution of leaf S and Se content in *msa1-1* X L*er*-0 F2 population. ICP-MS data is accessible using the digital object identifier (DOI) 10.4231/T99G5JRP (see http://dx.doi.org/).
(TIF)

S3 Fig Amino acid sequence alignment of SHM proteins and homology model of MSA1. (A) Amino acid sequence alignment of SHM proteins from *A*. *thaliana*, rice, human, rabbit, mouse, yeast and *E*. *coli*. Sequence alignment was performed by using Clustal W. Identical and similar residues are displayed in black or grey background. The mutated amino acid in MSA1 of the *MSA1* mutant was marked with red box. The binding sites of pyridoxal-5’-phosphate (PLP) and folate were marked with close red triangle and open blue triangle, respectively, based on the crystal structure of rabbit SHMT1 (OcSHMT1). The nuclear localization signal (NLS) of MSA1 was marked with magenta box. Protein sequences were extracted from GenBank. Arabidopsis (*Arabidopsis thaliana*): AtSHM1 (At4g37930), AtSHM2 (At5g26780.1), AtSHM3 (At4g32520), AtSHM4 (At4g13930), AtSHM5 (At4g13890), AtSHM6 (At1g22020), AtSHM7/AtMSA1 (At1g36370); Rice (*Oryza sativa*): OsSHM1 (Os03g0738400), OsSHM2 (AK101450), OsSHM3 (Os12g0409000), OsSHM5 (Os01g0874900), OsSHM6 (Os05g0429000); Human (*Homo sapiens*): HsSHMT1 (AAH07979.1), HsSHMT2 (AAH11911.1); Rabbit (*Oryctolagus cuniculus*): OcSHMT1 (P07511), OcSHMT2 (NP_001075874.1); Mouse (*Mus musculus*): MmSHMT1 (AAH26055.1), MmSHMT2 (AAH04825.1); Yeast (*Saccharomyces cerevisiae*): ScSHMT1 (P37292), ScSHMT2 (P37291); E. coli (*Escherichia coli*): EcSHMT (ACI79831.1). (B) Homology model of MSA1 generated by using rabbit SHMT1 (PDB ID: 1ls3) as a template. The Ribbon diagram only showed the residues from 136 to 591 of MSA1 with good alignment with the template. Black dash square indicated the region with mutated residue Ser186 in *msa1-1* mutant. (C) Close-up structure of the region with Ser186 and its neighbour residues in wild type MSA1 protein. (D) Close-up structure of the same region as in (B) with Ser186 mutation to Phe. The binding sites of pyridoxal-5’-phosphate (PLP) and folate were shown in magenta and blue, respectively, in (B) to (D). The Ser186 and Phe are shown in red. The green dash lines in (E) and (F) indicate H-bonds, and purple dash lines in (D) indicate steric hindrance.
(TIF)

S4 Fig Molecular characterization and the S and Se content of T-DNA insertion mutants of *MSA1*. (A) Gene structure of *MSA1*. Blue bars, grey lines and white bars represent exons, introns and untranslated region, respectively. Point mutation site was indicated by vertical lines. T-DNA insertion sites were indicated by triangles. The primers using for genotyping and qRT-PCR were shown as black arrows. (B) Genotyping T-DNA insertion lines. Gene specific primers (FP and RP) and left border primer of the T-DNA insertion (LB) were used. (C) Quantification of expression of *MSA1* in two T-DNA lines by qRT-PCR. The expression level was normalized to the internal control gene *UBQ10*, and relative expression level was presented as 2^-ΔΔCt^ with WT as reference. Data were shown as means ± SD (*n* = 3). (D, E) S and Se content in the leaf of WT, *msa1-1* and T-DNA insertion lines. Data are presented as means ± SD (*n =* 11 or 12). Columns with different letters indicate significant difference (*P* ≤ 0.01, LSD test). DW, dry weight.
(TIF)

S5 Fig Subcellular localization of MSA1. (A) Nuclear localization of MSA1 was not affected by S deficiency. Transgenic plants harboring wild-type MSA1 (GFP-MSA1w) and mutated MSA1 (GFP-MSA1m) were grown on S sufficient media (1500μM, S1500) or S deficient media (0μM, S0). The nucleus was stained by DAPI. Bar, 10μm. (B) Subcellular localization of MSA1 in the leaf. Transgenic plants harboring wild-type MSA1 (GFP-MSA1w) and mutated MSA1 (GFP-MSA1m) were grown on half-strength MS media for 5 days. The nucleus were stained by DAPI. Auto fluorescence indicates chloroplast. Bar, 10μm.
(TIF)

S6 Fig MSA1 is catalytically inactive *in vitro*. (A) The SHM activity of purified recombinant SHM proteins. SHM activity was determined using ^14^C-labeled Ser and monoglutamylated THF as substrates. pCal-n and pHUE are the empty vectors for expressing SHM1 and 2, and MSA1, respectively. (B) MSA1 failed to complement an *E*. *coli* glycine auxotroph (GS245(DE3)pLysS; *shmt*-). Growth of various strains in liquid media supplemented with different concentrations of IPTG for induction. 50 μg mL^-1^ glycine was added as control. OD630 was measured after 24 hours incubation. Bacteria SHMT was used as the positive control. Data in (A) and (B) are presented as means ± SD (*n* = 3). Columns with different letters in (A) indicate significant difference (*P* ≤ 0.01, least significant difference test).
(TIF)

S7 Fig Normalized DNA methylation level on CG, CHG and CHH contexts in the shoot and root of WT and *msa1-1* on chromosome 2 to 5. DNA methylation level was calculated by the density of methylated C in each 100 kb windows and the highest density windows in each contexts was designated as 100%.
(TIF)

S8 Fig Numbers of overlapping DMRs which were differentially methylated on gene body, 2 kb upstream, or 2 kb downstream flanks. Numbers of overlapping DMRs were shown separately in hyper-methylated in shoots and hypo-methylated in roots of WT and *msa1-1*.
(TIF)

S9 Fig Expression of genes involved in sulphate uptake, sulphur metabolism and metabolism of sulphated compounds in the *msa1-1* mutant. Total RNA was isolated from the shoot and root of WT and *msa1-1* mutant grown in soil for five weeks and the transcript level was determined by quantitative RT-PCR. The expression level was normalized to the internal control gene *UBQ10*, and presented as 2^(deltaCt) with means ± SD (*n =* 3). Single and double asterisks indicate values significantly different between WT and *msa1-1* mutant at *P* ≤ 0.05 and *P* ≤ 0.01, respectively (Student’s *t* test).
(PDF)

S10 Fig Genotyping *msa1-1 sultr1;1 sultr1;2* triple mutant and other control lines. The dCAPS primer 78730SNP2 was used to genotype the point mutation in *msa1-1* mutant. 08620LP2 and RP2 are *SULTR1;1*-specific primers, 78000LP1 and RP1 are *SULTR1;2*-specific primers, and XR2 and JL202 are T-DNA border primers. M, DNA ladder. The primer sequences are listed in S6 Table.
(TIF)

S1 Table Statistics of whole genome bisulfite sequencing samples. (XLSX)

S2 Table Cytosine methylation levels at CG, CHG, and CHH and total cytosine sites of the genes and transposon elements in WT and *msa1-1*. (XLSX)

S3 Table Global statistic of DMRs between WT and *msa1-1*. (XLSX)

S4 Table List of DMRs between shoots and roots of WT and *msa1-1*. (XLSX)

S5 Table List of hyper-DMGs and hypo-DMGs in shoots and roots. (XLSX)

S6 Table Gene ontology enrichment analysis of hyper-DMGs in hypo-DMGs in shoots and roots. (XLSX)

S7 Table List of DMGs that are responsive to sulphur starvation or involved in glucosinolate and anthocyanin metabolisms. (XLSX)

S8 Table Sequence-based methylation level of *SULTR1;1*, *SULTR1;2* and *APR3* in the shoot and root of WT and *msa1-1*. The position highlighted with blue and red colours indicate the location of the shoot and root DMRs, respectively. The position highlighted with grey colour indicate the location of primers used for the chop-PCR in Fig 6F. The sequence highlighted with yellow colour are SURE element.
(XLSX)

S9 Table The primers used in this study. (XLSX)
