# Supplementary figures and images for: Nuclear Localised MORE SULPHUR ACCUMULATION1 Epigenetically Regulates Sulphur Homeostasis in Arabidopsis thaliana
Source: PLoS Genet. 2016 Sep 13;12(9):e1006298. doi: 10.1371/journal.pgen.1006298 (PMC5021336; doi:10.1371/journal.pgen.1006298)

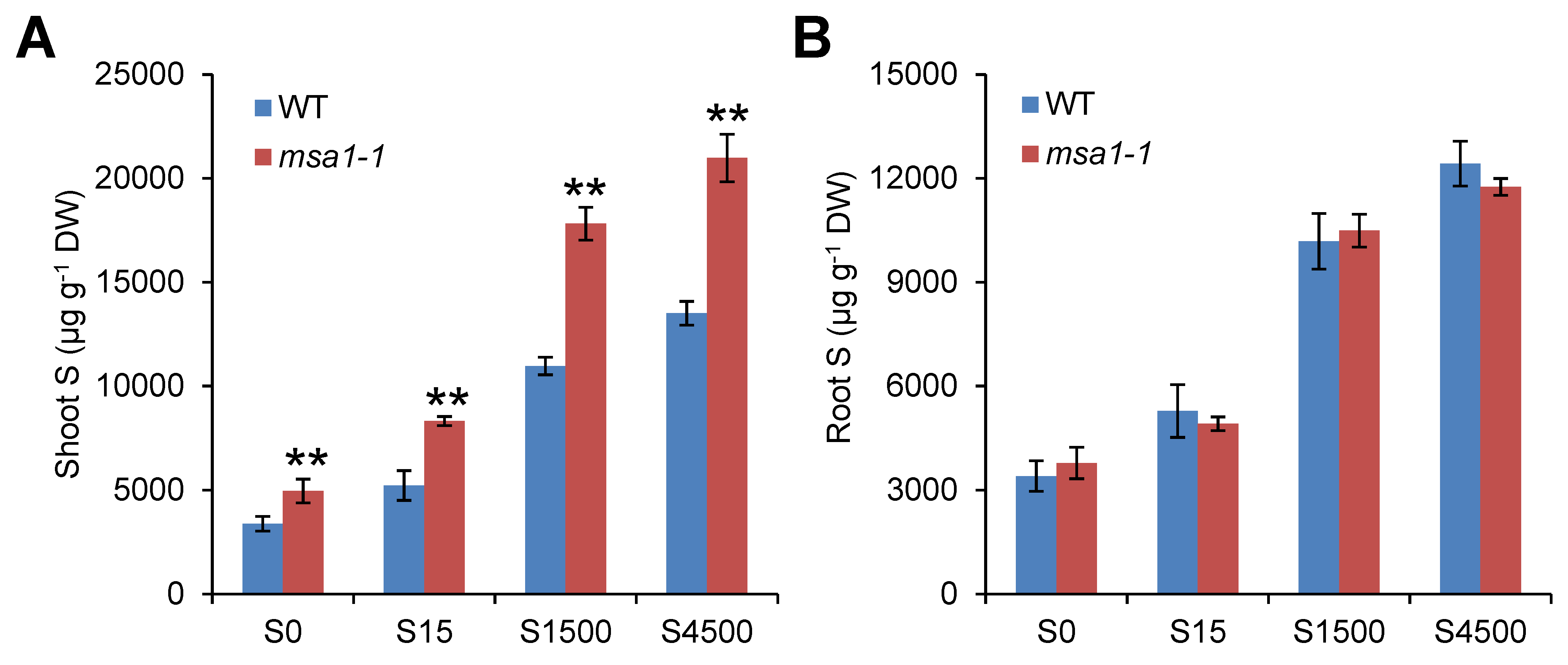

Supplement: S1 Fig — Plants were grown on MGRL agar media without sulphate (S0), with 15 μM (S15), 1500 μM (S1500) or 4500 μM (S4500) sulphate for two weeks. Total S in both shoots (A) and root (B) were determined. Data are presented as means ± SD (n = 6). Single and double asterisks indicate values significantly different between WT and msa1-1 mutant at P ≤ 0.05 and P ≤ 0.01, respectively (Student’s t test). DW, dry weight. (TIF) [file pgen.1006298.s001.tif]

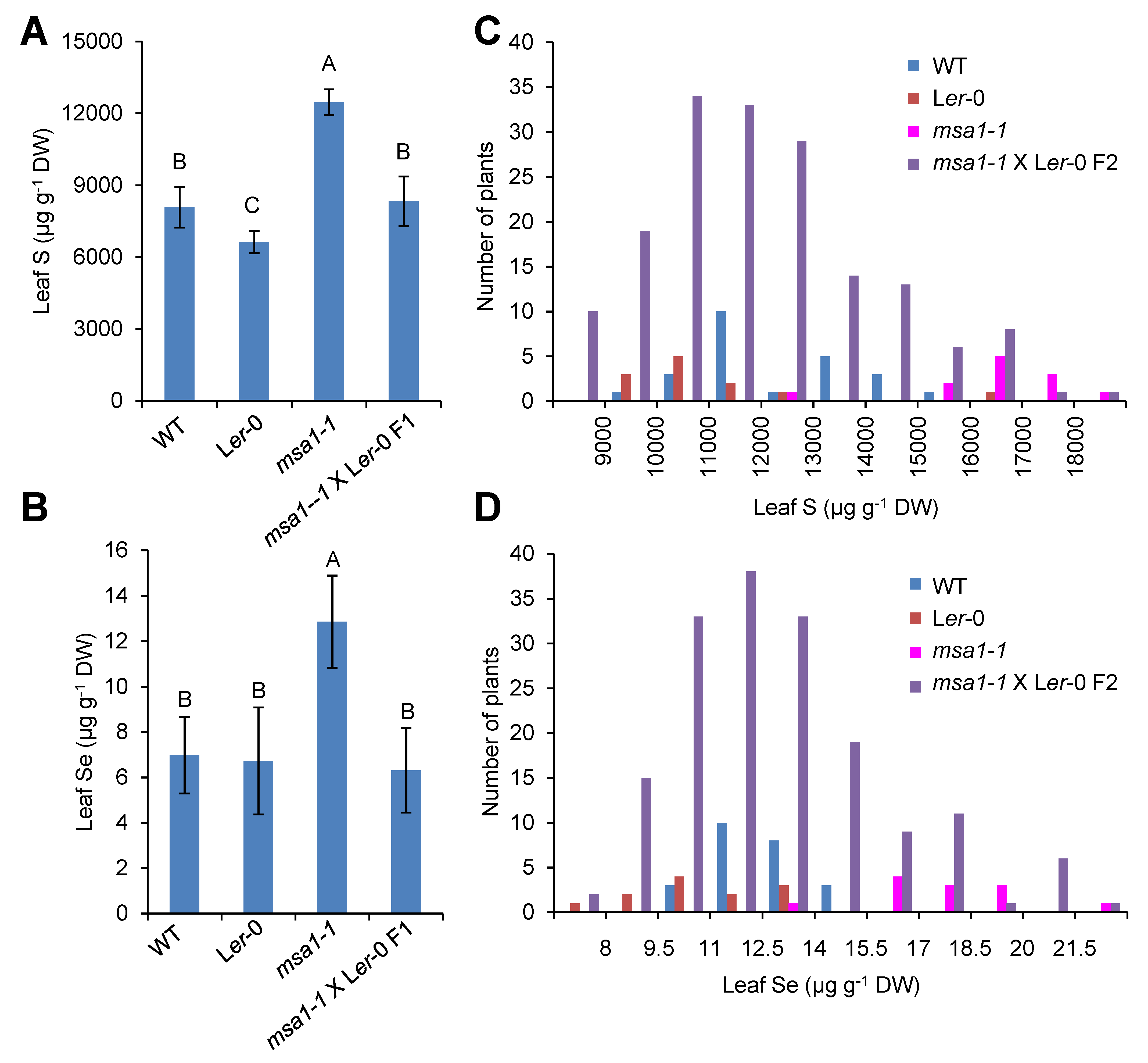

Supplement: S2 Fig — (A, B) S and Se content in the leaves of WT Col-0, Ler-0, msa1-1 and msa1-1 X Ler-0 F1 plants. Data are presented as means ± SD (n = 12). Columns with different letters indicate significant difference (P ≤ 0.01, least significant difference test). DW, dry weight. (C, D) The frequency distribution of leaf S and Se content in msa1-1 X Ler-0 F2 population. ICP-MS data is accessible using the digital object identifier (DOI) 10.4231/T99G5JRP (see http://dx.doi.org/). (TIF) [file pgen.1006298.s002.tif]

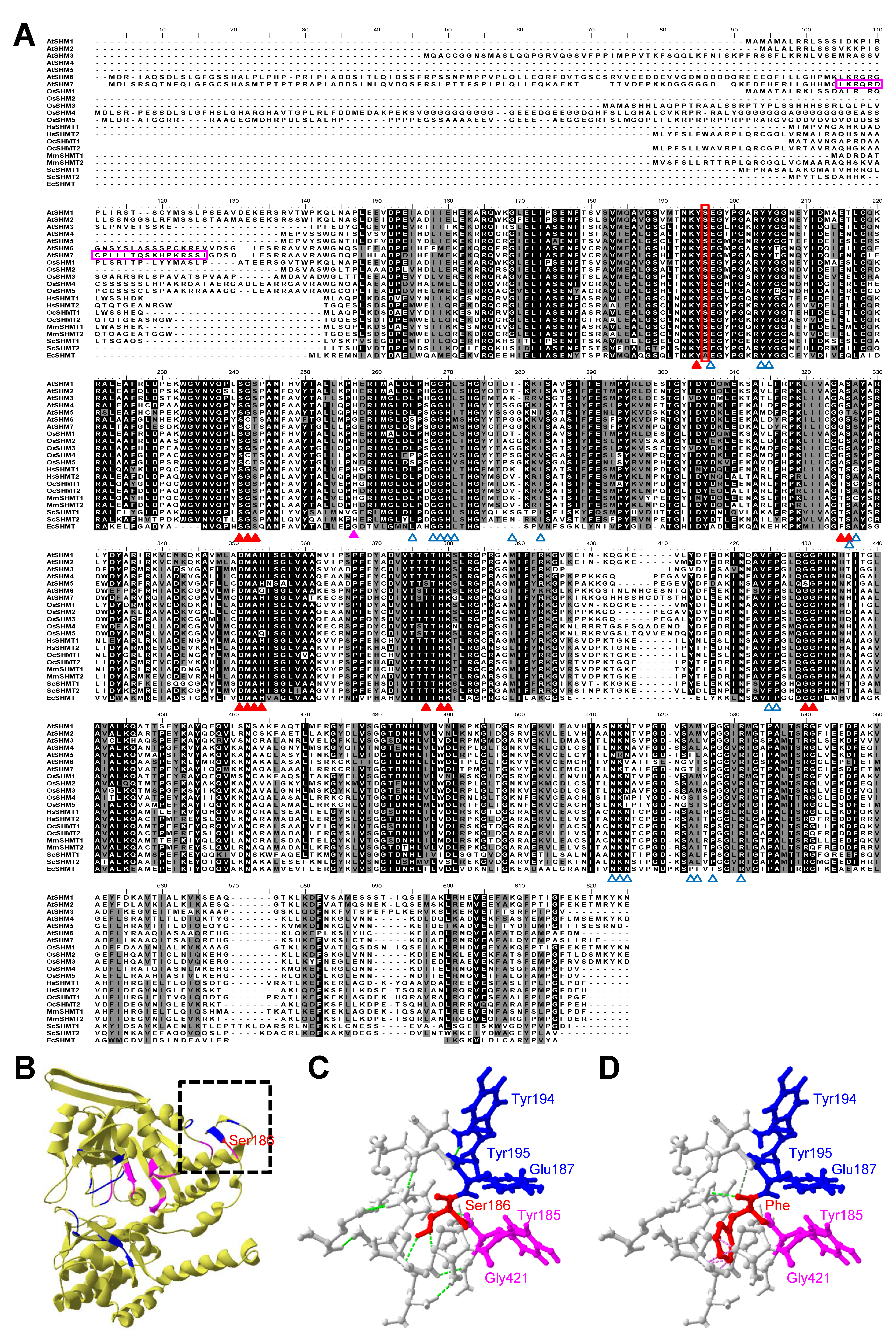

Supplement: S3 Fig — (A) Amino acid sequence alignment of SHM proteins from A. thaliana, rice, human, rabbit, mouse, yeast and E. coli. Sequence alignment was performed by using Clustal W. Identical and similar residues are displayed in black or grey background. The mutated amino acid in MSA1 of the MSA1 mutant was marked with red box. The binding sites of pyridoxal-5’-phosphate (PLP) and folate were marked with close red triangle and open blue triangle, respectively, based on the crystal structure of rabbit SHMT1 (OcSHMT1). The nuclear localization signal (NLS) of MSA1 was marked with magenta box. Protein sequences were extracted from GenBank. Arabidopsis (Arabidopsis thaliana): AtSHM1 (At4g37930), AtSHM2 (At5g26780.1), AtSHM3 (At4g32520), AtSHM4 (At4g13930), AtSHM5 (At4g13890), AtSHM6 (At1g22020), AtSHM7/AtMSA1 (At1g36370); Rice (Oryza sativa): OsSHM1 (Os03g0738400), OsSHM2 (AK101450), OsSHM3 (Os12g0409000), OsSHM5 (Os01g0874900), OsSHM6 (Os05g0429000); Human (Homo sapiens): HsSHMT1 (AAH07979.1), HsSHMT2 (AAH11911.1); Rabbit (Oryctolagus cuniculus): OcSHMT1 (P07511), OcSHMT2 (NP_001075874.1); Mouse (Mus musculus): MmSHMT1 (AAH26055.1), MmSHMT2 (AAH04825.1); Yeast (Saccharomyces cerevisiae): ScSHMT1 (P37292), ScSHMT2 (P37291); E. coli (Escherichia coli): EcSHMT (ACI79831.1). (B) Homology model of MSA1 generated by using rabbit SHMT1 (PDB ID: 1ls3) as a template. The Ribbon diagram only showed the residues from 136 to 591 of MSA1 with good alignment with the template. Black dash square indicated the region with mutated residue Ser186 in msa1-1 mutant. (C) Close-up structure of the region with Ser186 and its neighbour residues in wild type MSA1 protein. (D) Close-up structure of the same region as in (B) with Ser186 mutation to Phe. The binding sites of pyridoxal-5’-phosphate (PLP) and folate were shown in magenta and blue, respectively, in (B) to (D). The Ser186 and Phe are shown in red. The green dash lines in (E) and (F) indicate H-bonds, and purple dash lines in (D) indicate steric h [file pgen.1006298.s003.tif]

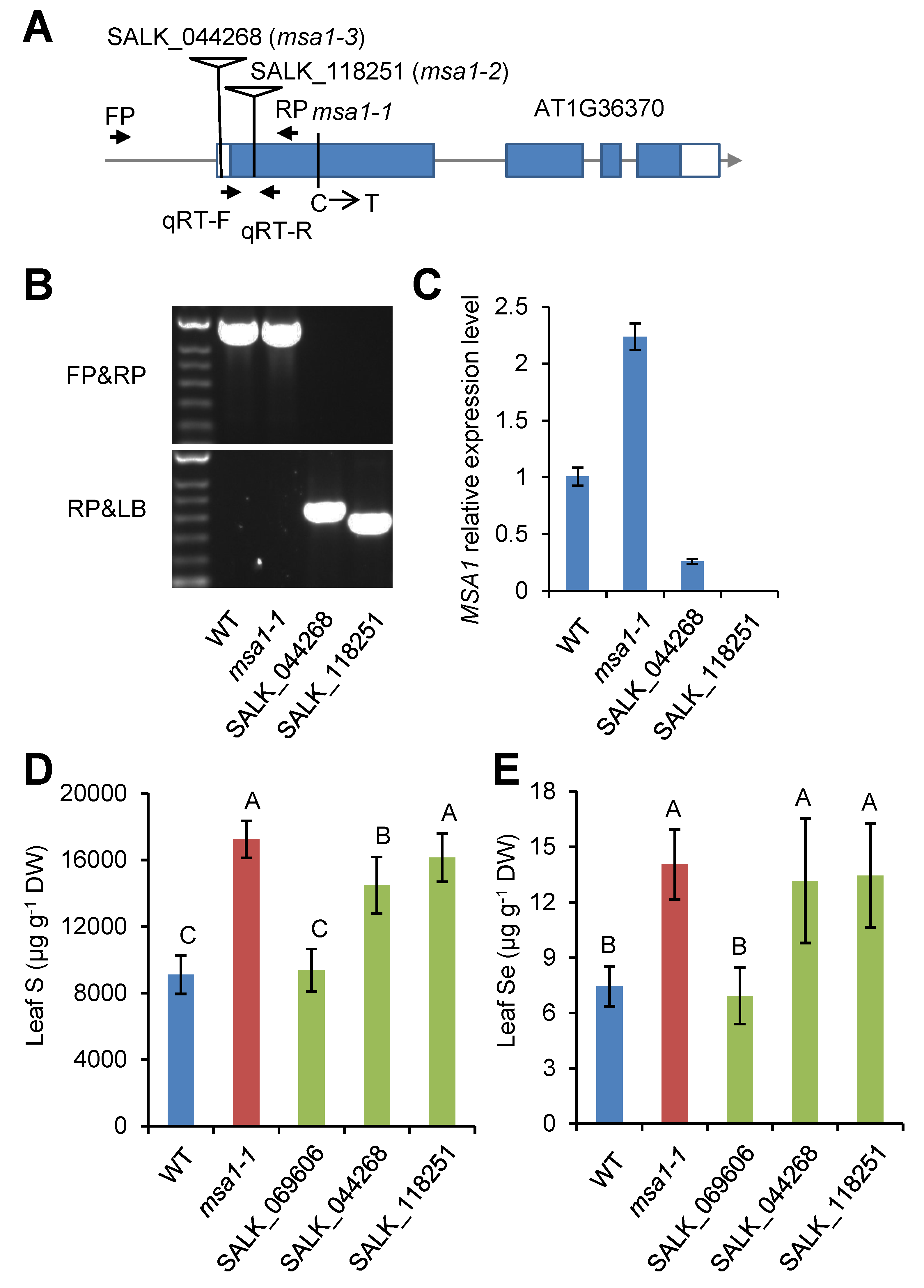

Supplement: S4 Fig — (A) Gene structure of MSA1. Blue bars, grey lines and white bars represent exons, introns and untranslated region, respectively. Point mutation site was indicated by vertical lines. T-DNA insertion sites were indicated by triangles. The primers using for genotyping and qRT-PCR were shown as black arrows. (B) Genotyping T-DNA insertion lines. Gene specific primers (FP and RP) and left border primer of the T-DNA insertion (LB) were used. (C) Quantification of expression of MSA1 in two T-DNA lines by qRT-PCR. The expression level was normalized to the internal control gene UBQ10, and relative expression level was presented as 2-ΔΔCt with WT as reference. Data were shown as means ± SD (n = 3). (D, E) S and Se content in the leaf of WT, msa1-1 and T-DNA insertion lines. Data are presented as means ± SD (n = 11 or 12). Columns with different letters indicate significant difference (P ≤ 0.01, LSD test). DW, dry weight. (TIF) [file pgen.1006298.s004.tif]

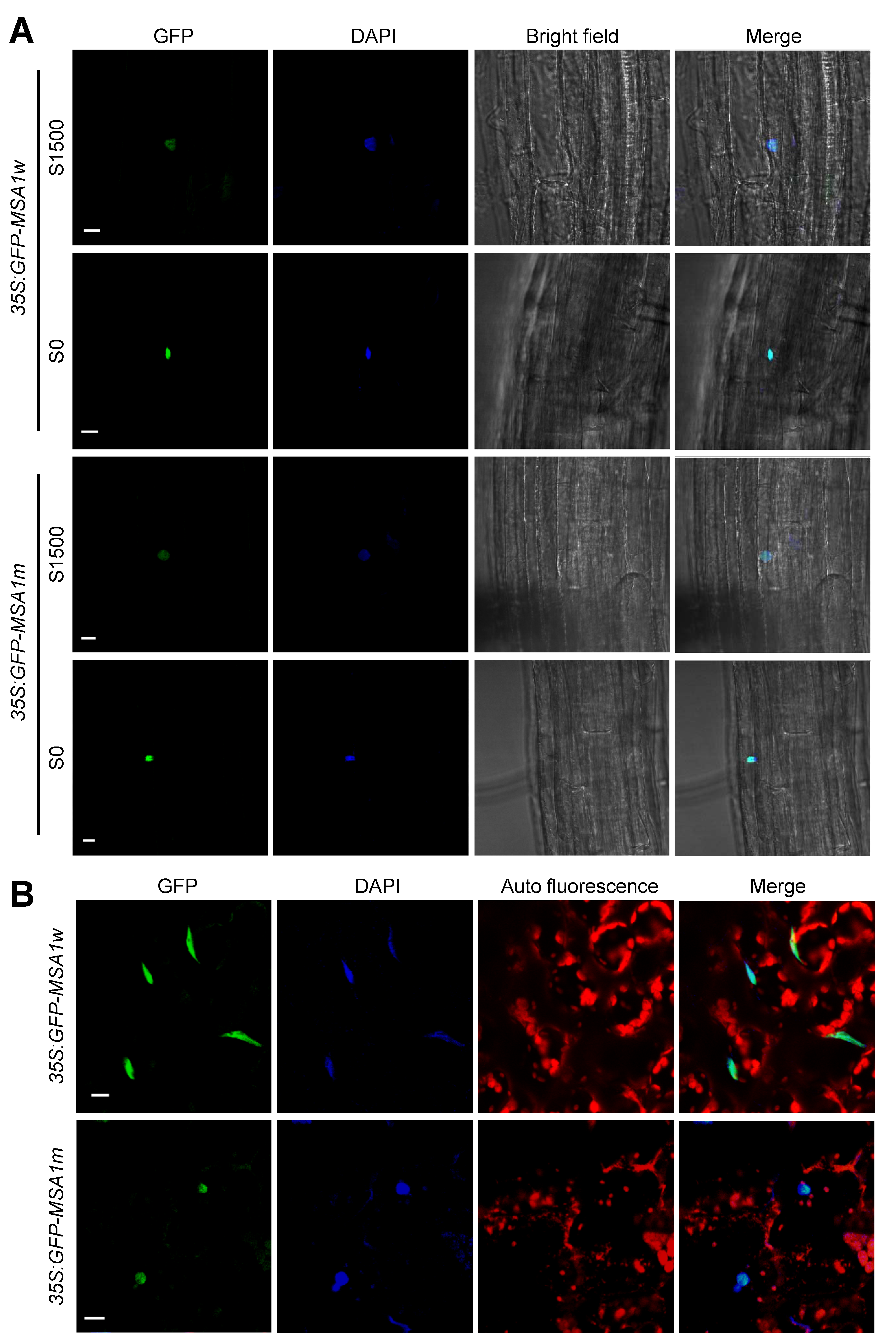

Supplement: S5 Fig — (A) Nuclear localization of MSA1 was not affected by S deficiency. Transgenic plants harboring wild-type MSA1 (GFP-MSA1w) and mutated MSA1 (GFP-MSA1m) were grown on S sufficient media (1500μM, S1500) or S deficient media (0μM, S0). The nucleus was stained by DAPI. Bar, 10μm. (B) Subcellular localization of MSA1 in the leaf. Transgenic plants harboring wild-type MSA1 (GFP-MSA1w) and mutated MSA1 (GFP-MSA1m) were grown on half-strength MS media for 5 days. The nucleus were stained by DAPI. Auto fluorescence indicates chloroplast. Bar, 10μm. (TIF) [file pgen.1006298.s005.tif]

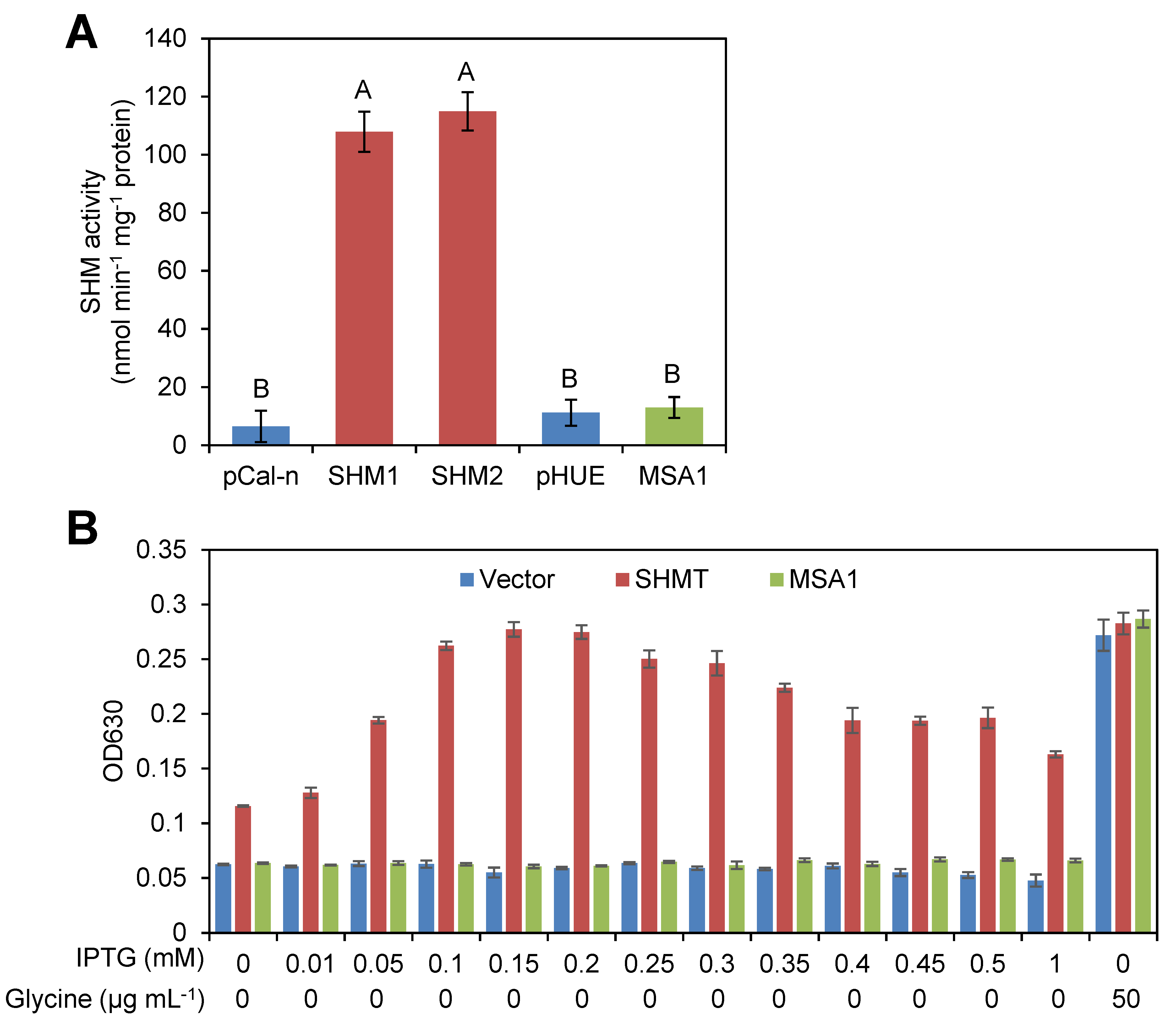

Supplement: S6 Fig — (A) The SHM activity of purified recombinant SHM proteins. SHM activity was determined using 14C-labeled Ser and monoglutamylated THF as substrates. pCal-n and pHUE are the empty vectors for expressing SHM1 and 2, and MSA1, respectively. (B) MSA1 failed to complement an E. coli glycine auxotroph (GS245(DE3)pLysS; shmt-). Growth of various strains in liquid media supplemented with different concentrations of IPTG for induction. 50 μg mL-1 glycine was added as control. OD630 was measured after 24 hours incubation. Bacteria SHMT was used as the positive control. Data in (A) and (B) are presented as means ± SD (n = 3). Columns with different letters in (A) indicate significant difference (P ≤ 0.01, least significant difference test). (TIF) [file pgen.1006298.s006.tif]

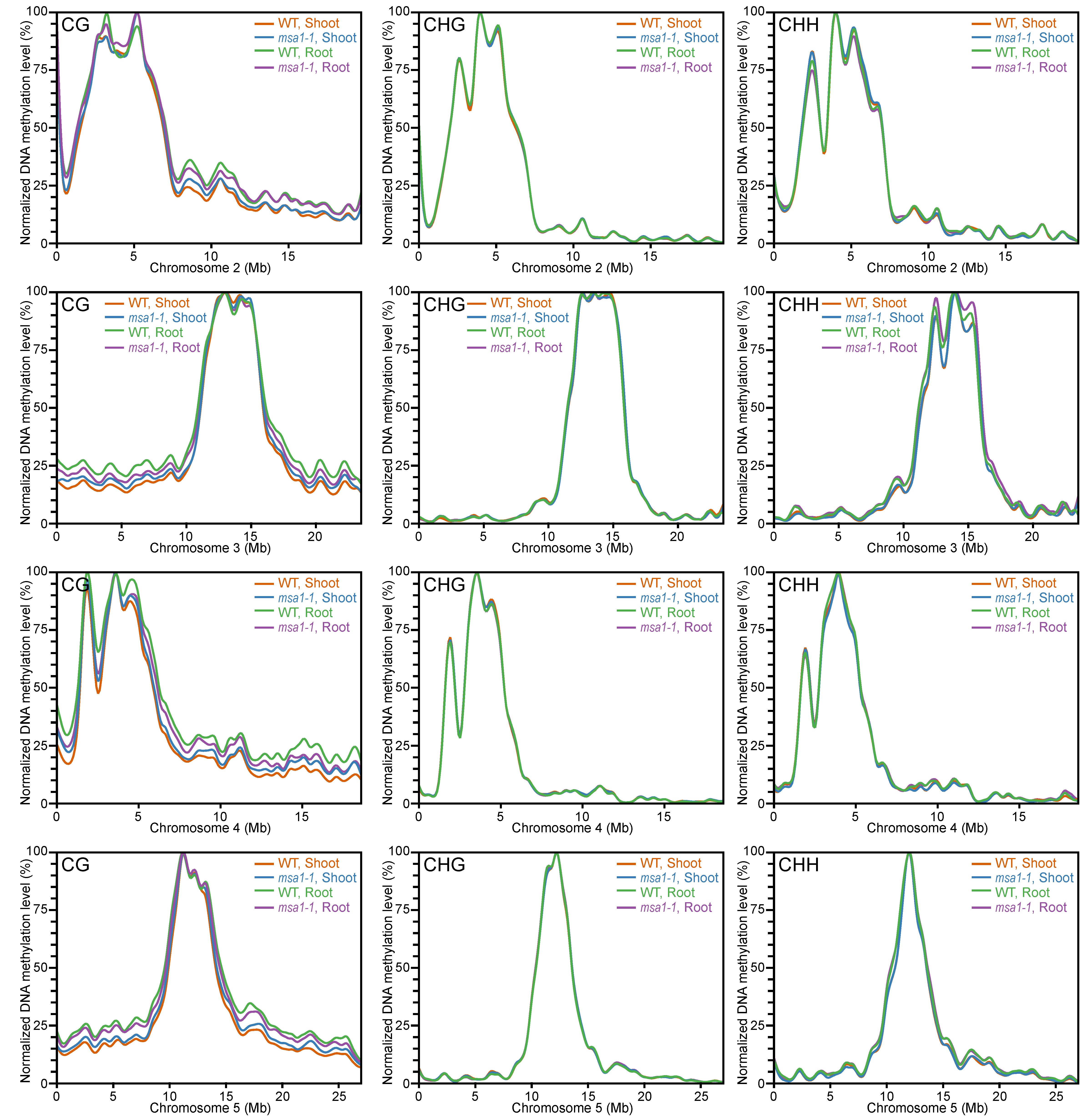

Supplement: S7 Fig — DNA methylation level was calculated by the density of methylated C in each 100 kb windows and the highest density windows in each contexts was designated as 100%. (TIF) [file pgen.1006298.s007.tif]

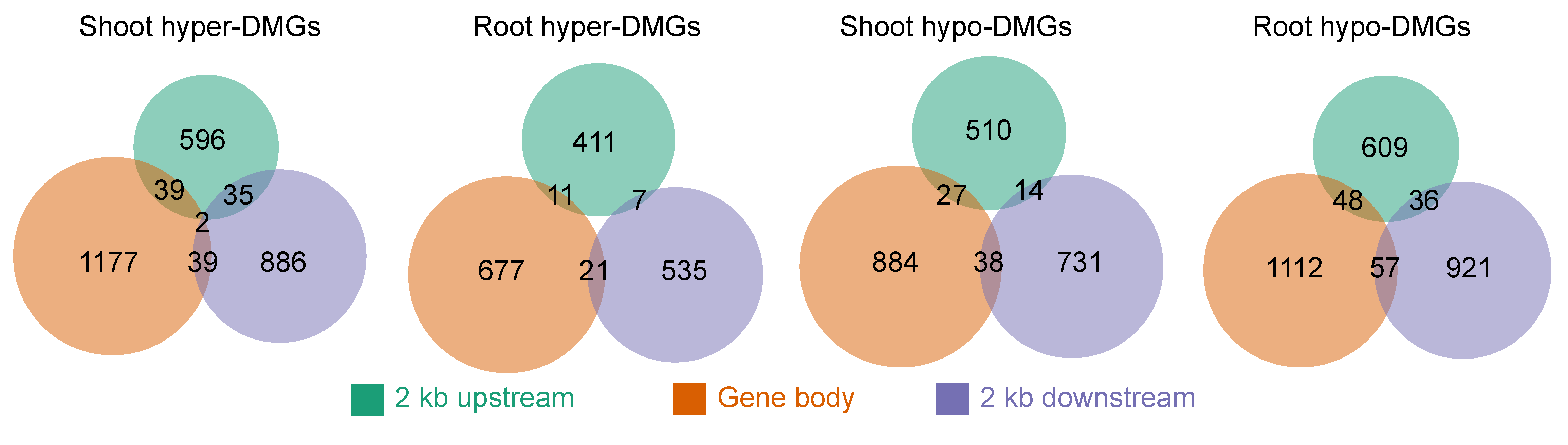

Supplement: S8 Fig — Numbers of overlapping DMRs were shown separately in hyper-methylated in shoots and hypo-methylated in roots of WT and msa1-1. (TIF) [file pgen.1006298.s008.tif]

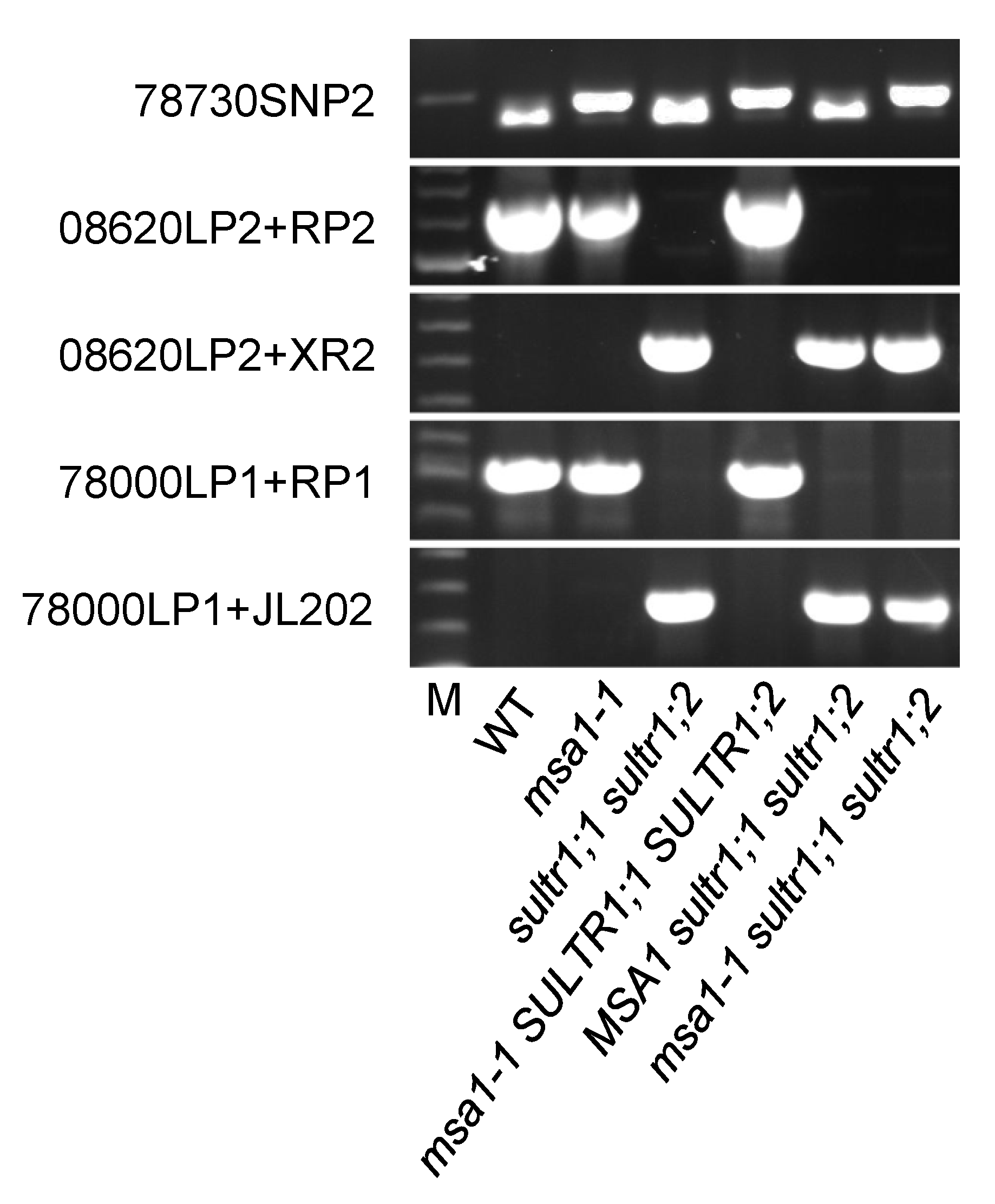

Supplement: S10 Fig — The dCAPS primer 78730SNP2 was used to genotype the point mutation in msa1-1 mutant. 08620LP2 and RP2 are SULTR1;1-specific primers, 78000LP1 and RP1 are SULTR1;2-specific primers, and XR2 and JL202 are T-DNA border primers. M, DNA ladder. The primer sequences are listed in S6 Table. (TIF) [file pgen.1006298.s010.tif]
