# Supplementary material for: Nuclear Localised MORE SULPHUR ACCUMULATION1 Epigenetically Regulates Sulphur Homeostasis in Arabidopsis thaliana
Source: PLoS Genet. 2016 Sep 13;12(9):e1006298. doi: 10.1371/journal.pgen.1006298 (PMC5021336; doi:10.1371/journal.pgen.1006298)
Supplement: S9 Fig — Total RNA was isolated from the shoot and root of WT and msa1-1 mutant grown in soil for five weeks and the transcript level was determined by quantitative RT-PCR. The expression level was normalized to the internal control gene UBQ10, and presented as 2^(deltaCt) with means ± SD (n = 3). Single and double asterisks indicate values significantly different between WT and msa1-1 mutant at P ≤ 0.05 and P ≤ 0.01, respectively (Student’s t test). (PDF) [file pgen.1006298.s009.pdf]

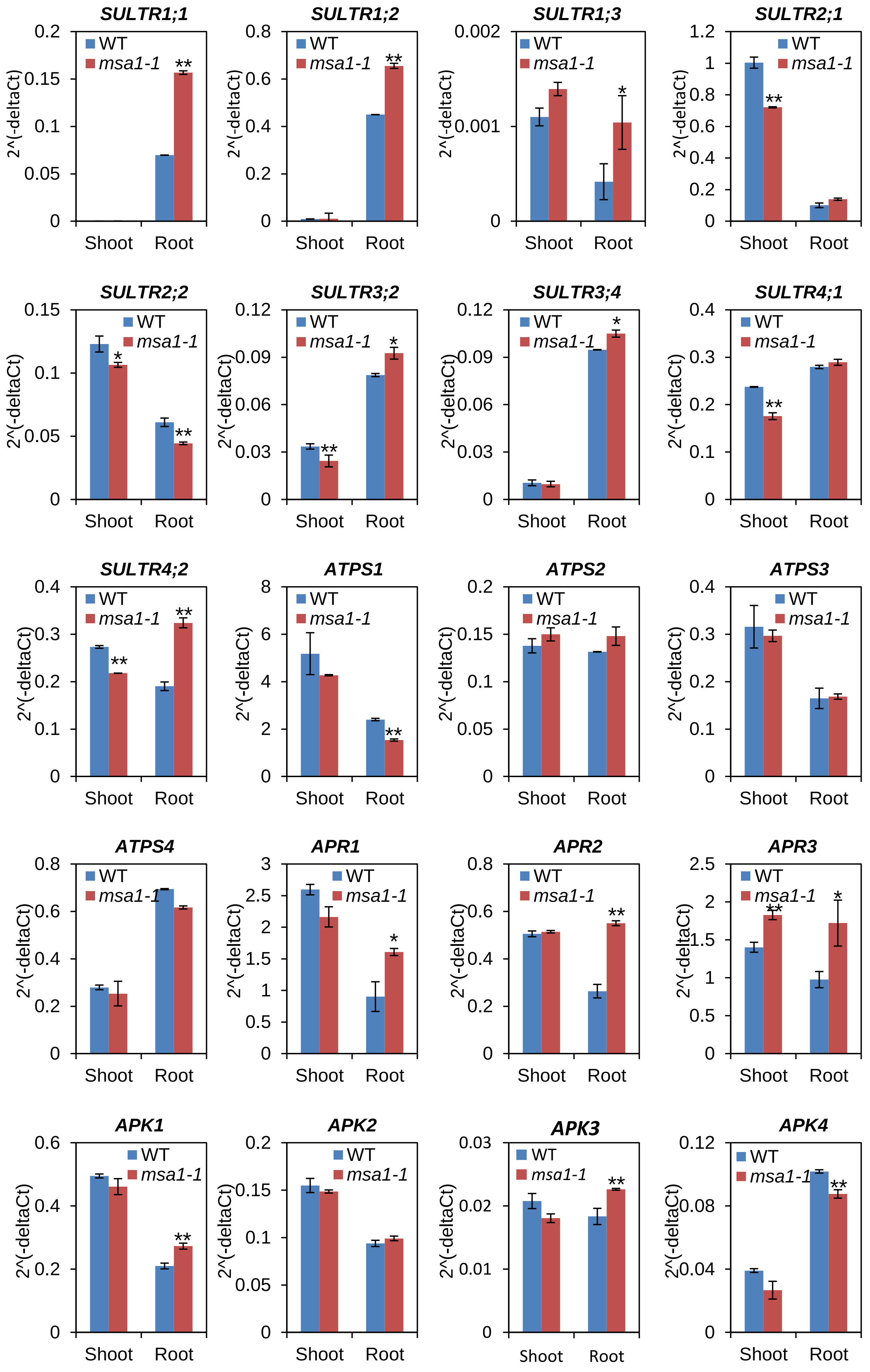
SULTR1;1
SULTR1;2
SULTR1;3
SULTR2;1
SULTR2;2
SULTR3;2
SULTR3;4
SULTR4;1
SULTR4;2
ATPS1
ATPS2
ATPS3
ATPS4
APR1
APR2
APR3
APK1
APK2
APK3
APK4
WT
msa1-1
2^(-deltaCt)
Shoot
Root
*
**

S9 Fig continued

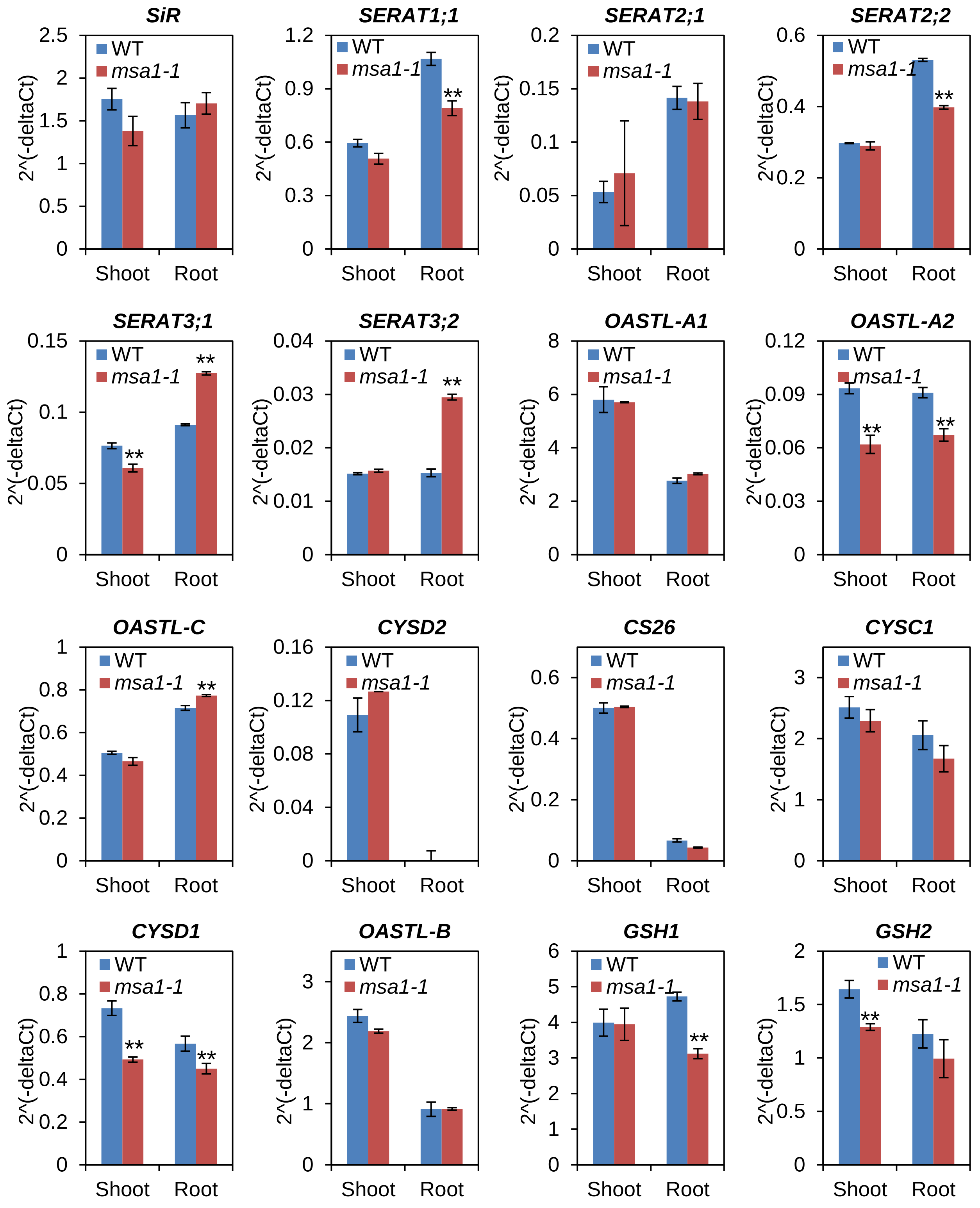

S9 Fig. Expression of genes involved in sulphate uptake, sulphur metabolism and metabolism of sulphated compounds in the *msa1-1* mutant. Total RNA was isolated from the shoot and root of WT and *msa1-1* mutant grown in soil for five weeks and the transcript level was determined by quantitative RT-PCR. The expression level was normalized to the internal control gene *UBQ10*, and presented as 2^(deltaCt) with means ± SD (n = 3). Single and double asterisks indicate values significantly different between WT and *msa1-1* mutant at $P \leq 0.05$ and $P \leq 0.01$, respectively (Student's t test).
